# Supplementary material for: Dual clathrin and integrin signaling systems regulate growth factor receptor activation
Source: Nat Commun. 2022 Feb 16;13:905. doi: 10.1038/s41467-022-28373-x (PMC8850434; doi:10.1038/s41467-022-28373-x)
Supplement: Supplementary file 1 — Supplementary Information [file 41467_2022_28373_MOESM1_ESM.pdf]

Supplementary Information

Dual clathrin and integrin signaling systems regulate growth factor receptor activation

Marco A. Alfonzo-Mendez[1], Kem A. Sochacki[1], Marie-Paule Strub[1], Justin W. Taraska[1*]

1.Biochemistry and Biophysics Center, National Heart, Lung, and Blood Institute, National Institutes of Health, 50 South Drive, Building 50, Bethesda, MD 20892

*Correspondence should be addressed to Justin W. Taraska (justin.taraska@nih.gov)

Figure	
Supplementary Figure 1	EGF increases the density of clathrin at the plasma membrane
Supplementary Figure 2	Effect of EGF on the ultrastructure of clathrin at the dorsal plasma membrane
Supplementary Figure 3	EGFR, Src and β5-integrin siRNAs validation
Supplementary Figure 4	Morphometric analysis of PREM images in Figures 2-4
Supplementary Figure 5	Morphometric analysis of PREM images of cells treated with different drugs and siRNAs
Supplementary Figure 6	Role of actin cytoskeleton in flat clathrin lattice expansion
Supplementary Figure 7	Flat clathrin lattice expansion is ERK independent
Supplementary Figure 8	Over-expressed EGFR-GFP correlates with clathrin after EGF stimulation
Supplementary Figure 9	Time course of Src correlation with clathrin after EGF stimulation
Supplementary Figure 10	Correlative TIRF and electron microscopy of β5-integrin
Supplementary Figure 11	Differential location of EGFR and Src in β5-integrin enriched structures
Supplementary Figure 12	Correlation of clathrin-coated structures with different integrins
Supplementary Figure 13	*In silico* analysis of β5-integrin
Supplementary Figure 14	EGF increases cell adhesion at clathrin-coated sites
Supplementary Figure 15	Flat clathrin lattices partition sustained signals at the plasma membrane
Table	
Supplementary Table 1	Table of information about plasmids generated in our study
Supplementary Table 2	Table showing primers used for the identification of the plasmids in our study

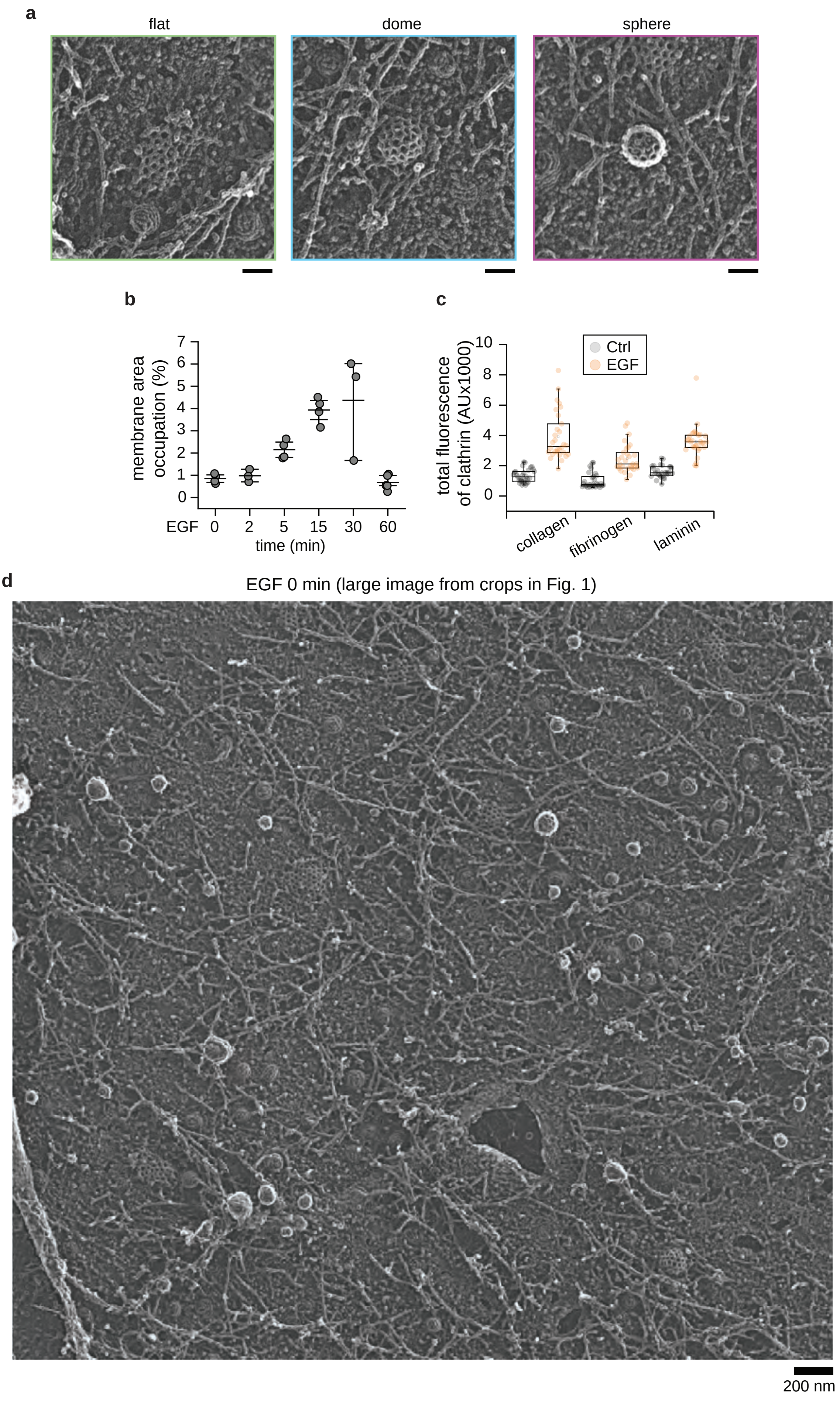
a
flat
dome
sphere
b
membrane area occupation (%)
7
6
5
4
3
2
1
0
EGF 0 2 5 15 30 60
time (min)
c
total fluorescence of clathrin (AUx1000)
10
8
6
4
2
0
Ctrl
EGF
collagen
fibrinogen
laminin
d
EGF 0 min (large image from crops in Fig. 1)
200 nm

EGF 2 min (large image from crops in Fig. 1)

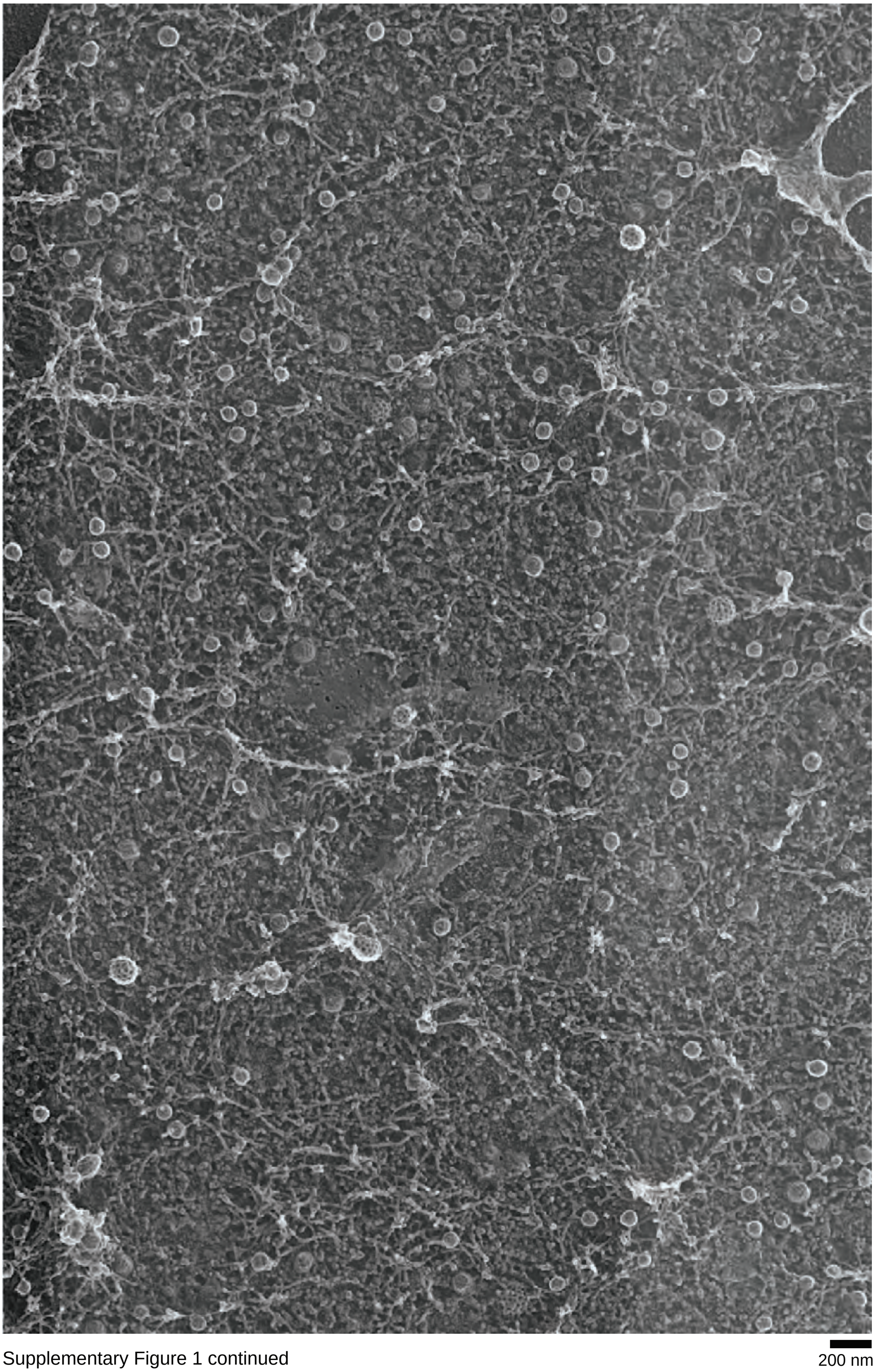

Supplementary Figure 1 continued

EGF 5 min (large image from crops in Fig. 1)

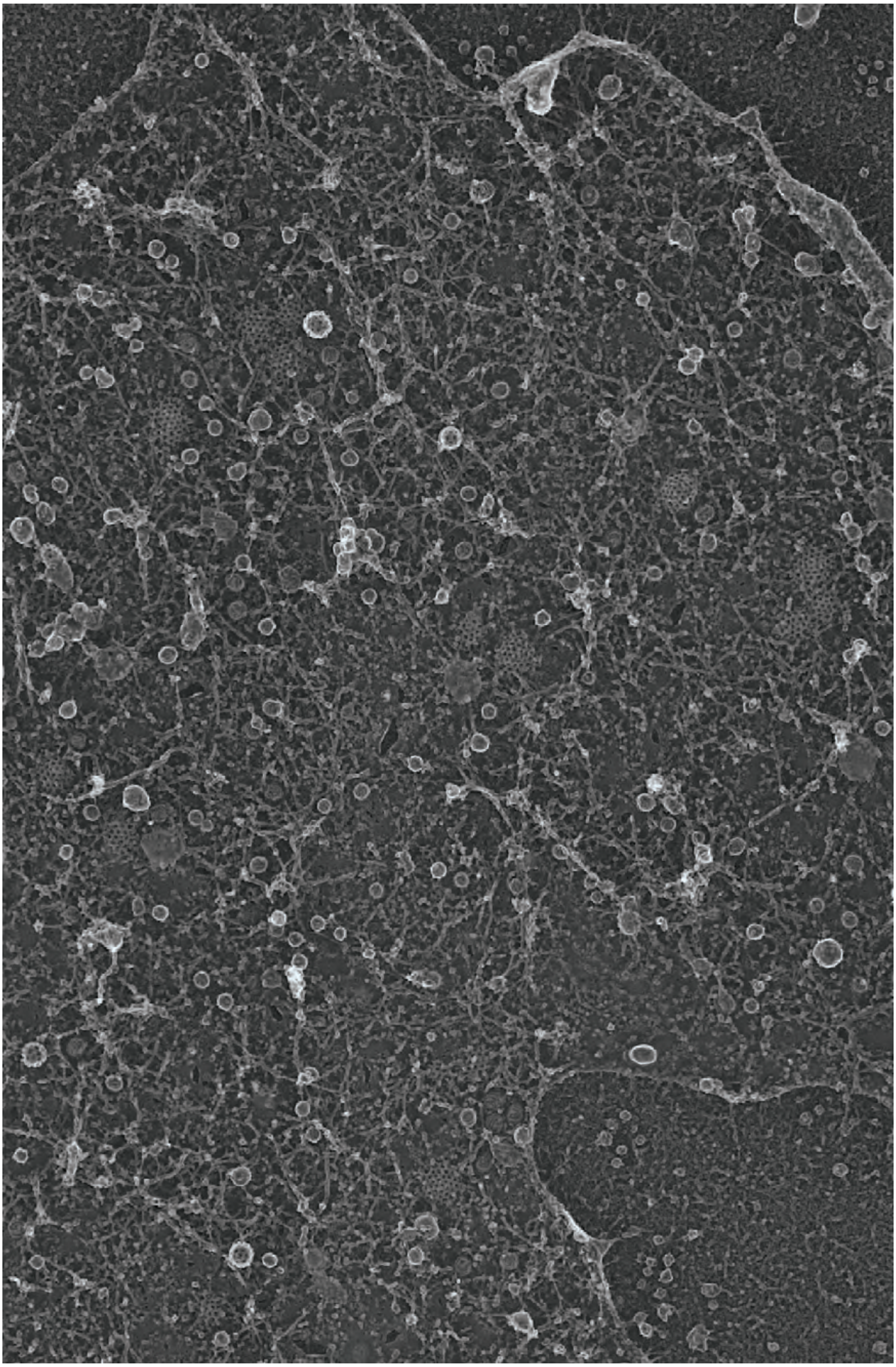

Supplementary Figure 1 continued

200 nm

EGF 15 min (large image from crops in Fig. 1)

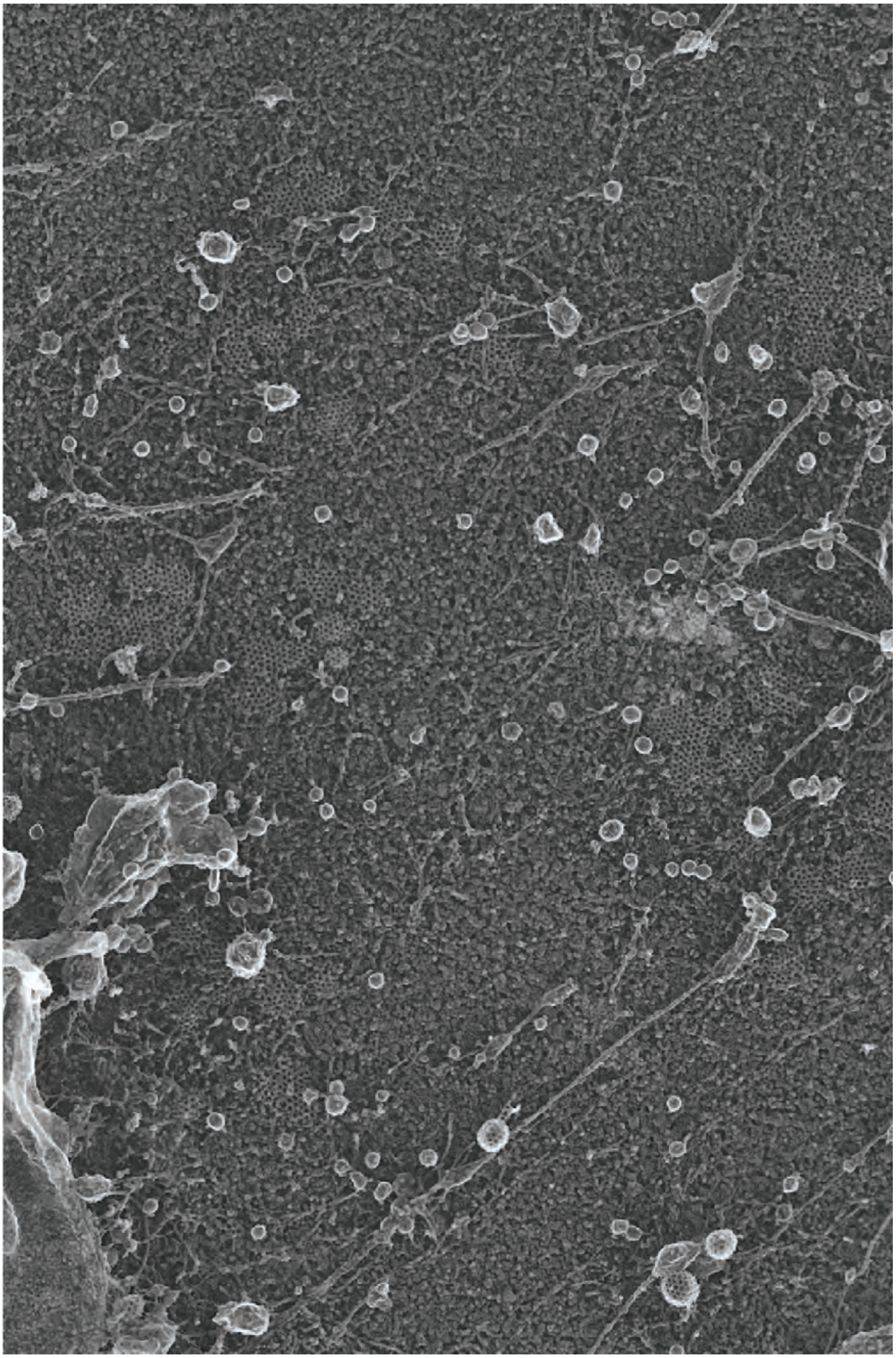

Supplementary Figure 1 continued

EGF 30 min (large image from crops in Fig. 1)

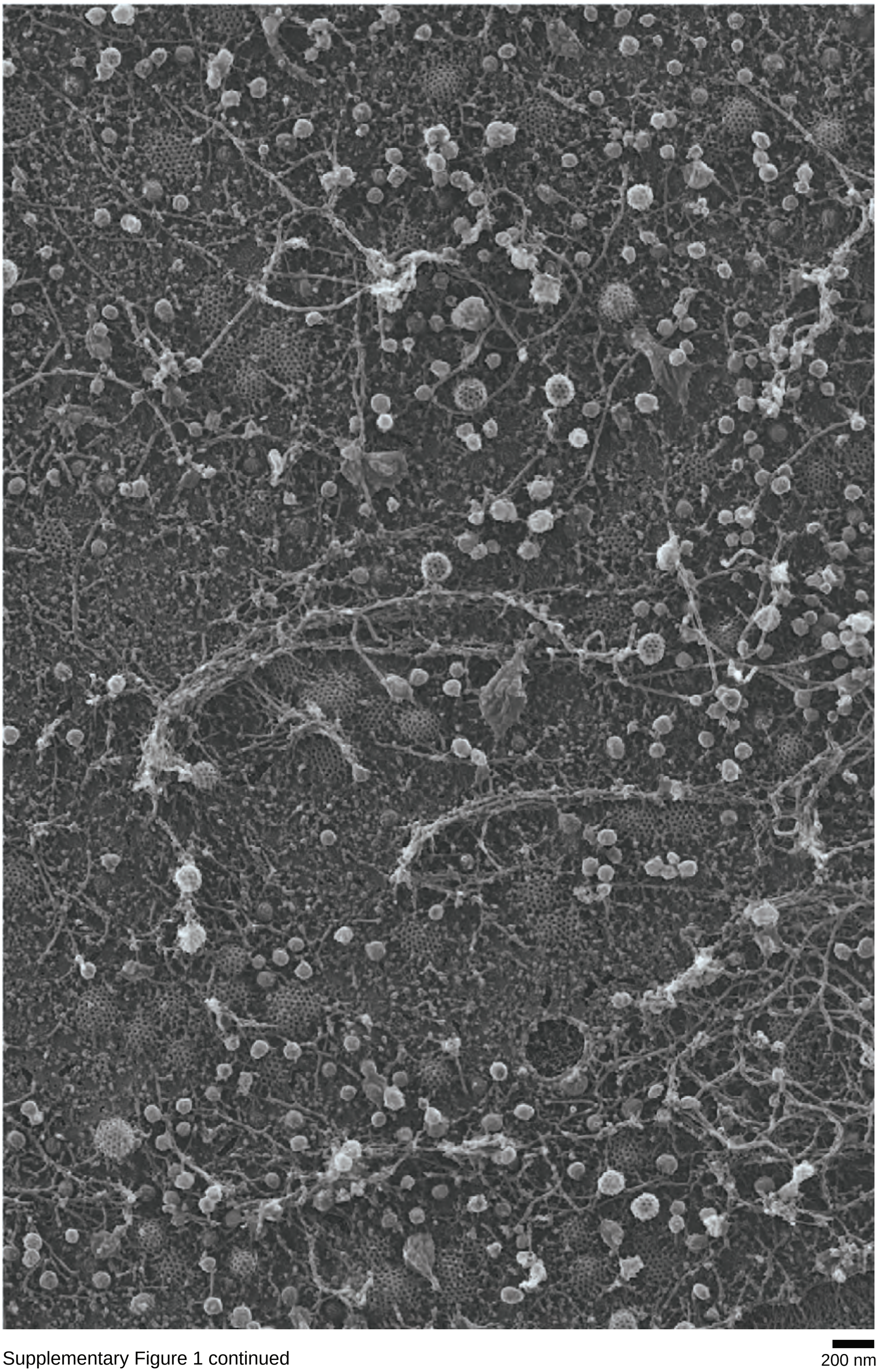

Supplementary Figure 1 continued

EGF 60 min (large image from crops in Fig. 1)

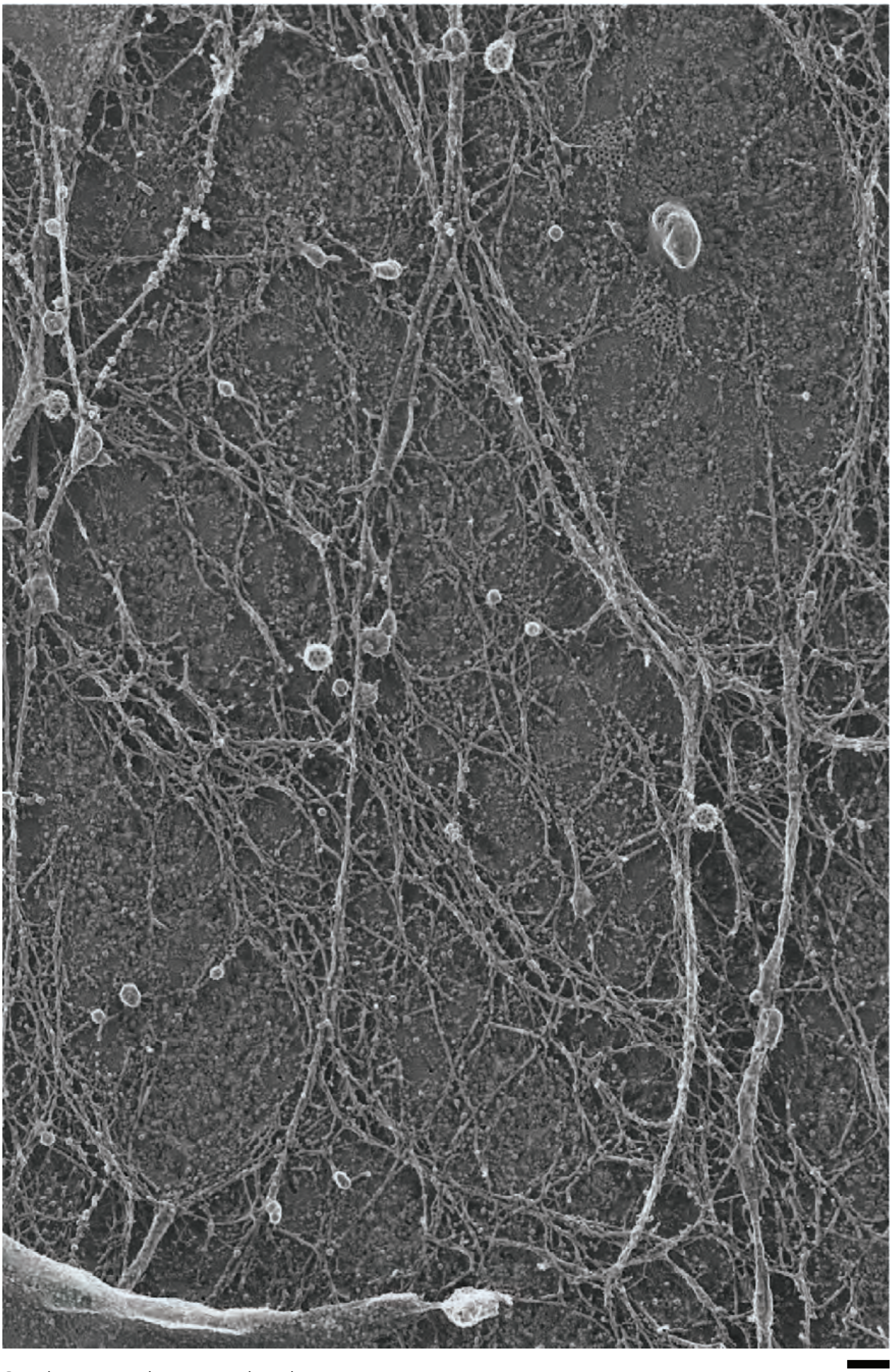

Supplementary Figure 1 continued

Supplementary Figure 1. EGF increases the density of clathrin at the plasma membrane. **a,** Representative PREM images of the flat (green), dome (blue), and sphere (magenta) clathrin-coated structures (CCSs) segmented in Fig. 1. Scale bars are 100 nm. **b,** Morphometric analysis of the percentage of plasma membrane (PM) area occupation for all clathrin-coated structures (CCSs) in PREM images of control (Ctrl) HSC3-EGFR-GFP cells or treated with 50 ng/mL EGF for 2, 5, 15, 30 and 60 min from Figure 1. I-shaped box plots show median extended from 25th to 75th percentiles, and minimum and maximum data point whiskers with a coefficient value of 1.5. 0 min: N_{cells}=4; 2 min: N_{cells}=3; 5 min: N_{cells}=4; 15 min: N_{cells}=4; 30 min: N_{cells}=3; 60 min: N_{cells}=5. **c,** Fluorescence intensity measurements of the signal from clathrin heavy chain in cells grown on collagen-, fibrinogen-, and laminin-coated coverslips. Control (Ctrl) unroofed HSC3-EGFR-GFP cells or treated with 50 ng/mL EGF were immunolabeled with anti-clathrin heavy chain coupled to Alexa 647. Collagen Ctrl: N_{cells}=30 cells; collagen EGF: N_{cells}=30; laminin Ctrl: N_{cells}=30 cells; laminin EGF: N_{cells}=30; fibrinogen Ctrl: N_{cells}=30 cells; fibrinogen EGF: N_{cells}=30. **d,** Original PREM images of cells from which the cropped images in Figure 1 were derived. PREM images of control (Ctrl) HSC3-EGFR-GFP cells, stimulated with 50 ng/mL EGF for 0, 2, 5, 15, 30 and 60 min. Scale bars are 200 nm. N= 2 biologically independent experiments in (**b, d**) and 3 in (**c**).

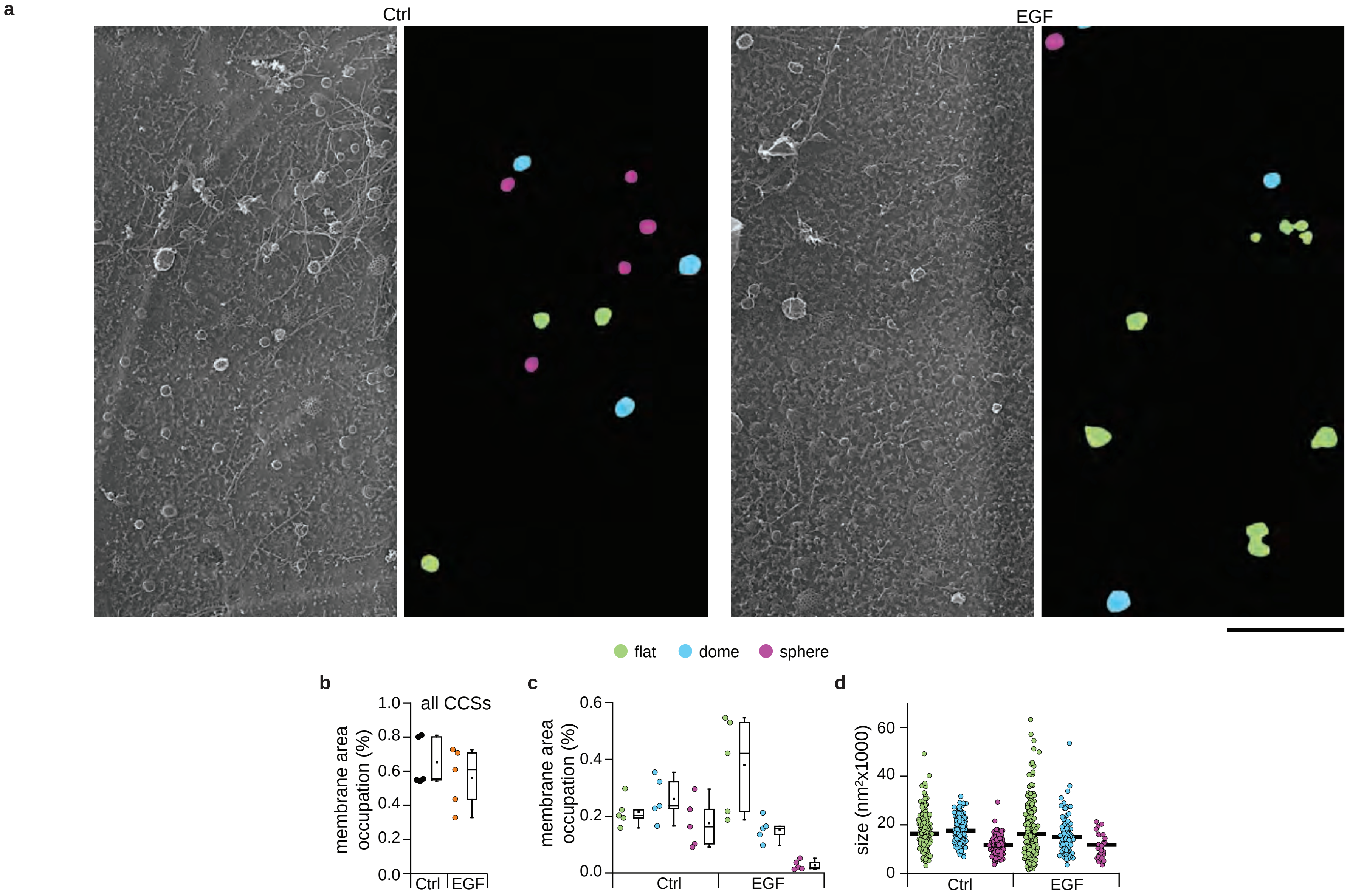

Supplementary Figure 2. Effect of EGF on the ultrastructure of clathrin at the dorsal plasma membrane. a, Representative PREMs and masks after segmentation of control HSC3-EGFR-GFP cells (Ctrl) or treated with 50 ng/mL EGF (EGF) for 15 min. Flat, dome, and sphere clathrin-coated structures (CCSs) are shown in green, blue, and magenta, respectively. Scale bar is 1 μm. **b,** Morphometric analysis of the percentage of dorsal plasma membrane (PM) area occupation for all clathrin-coated structures (CCSs) or (**c**) flat, dome and sphere CCSs. Box plots show median extended from 25th to 75th percentiles, black squares show mean, and minimum and maximum data point whiskers with a coefficient value of 1.5. **d,** Morphometric analysis of the size of flat, dome, and sphere CCSs of cells treated as indicated in (a). Dot plots show every structure segmented; the bar is the median. Ctrl: N_{flat}=191, N_{dome}=233, N_{sphere}=270, N_{cells}=5; EGF: N_{flat}=262, N_{dome}=132, N_{sphere}=28, N_{cells}=5. Number of biologically independent experiments with consistent results=2 (**b-d**).

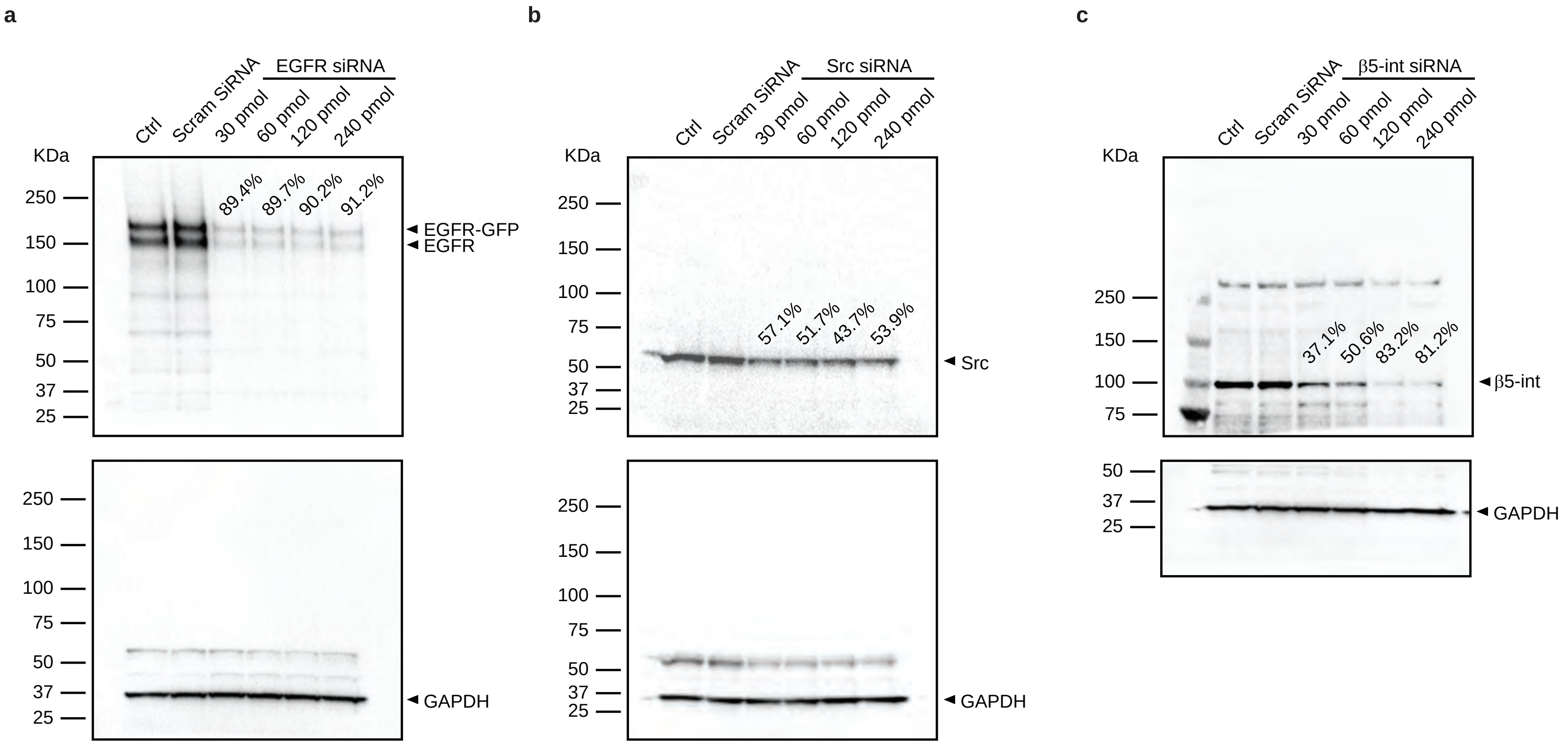

Supplementary Figure 3. EGFR, Src and β5-integrin siRNAs validation. Western blot analysis of extracts from untransfected HSC3-EGFR-GFP cells (Ctrl), cells transfected with 30 pmol of untargeted scramble siRNA (Scram siRNA), or increasing concentrations of targeted (**a**) EGFR, (**b**) Src and (**c**) β5-integrin siRNAs, respectively. GAPDH immunoblotting is shown to verify equal loading. Arrowheads indicate bands for the corresponding proteins. The percentage of knock down efficiency was calculated by pixel densitometry and normalized to controls. N=2 biologically independent experiments with consistent results in (**a**-**c**).

Ctrl (Large image from crops in Fig. 2)

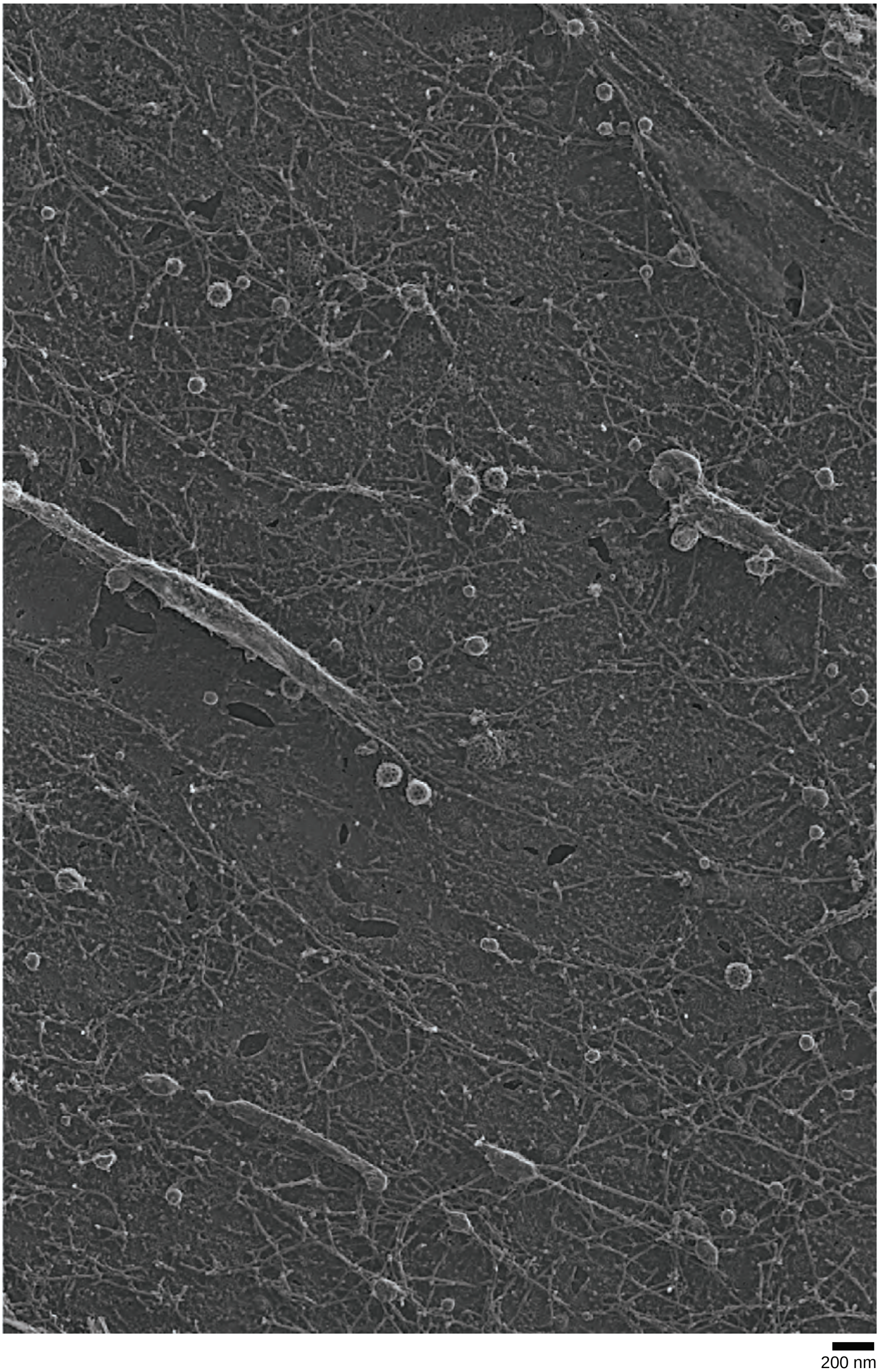

EGF (Large image from crops in Fig. 2)

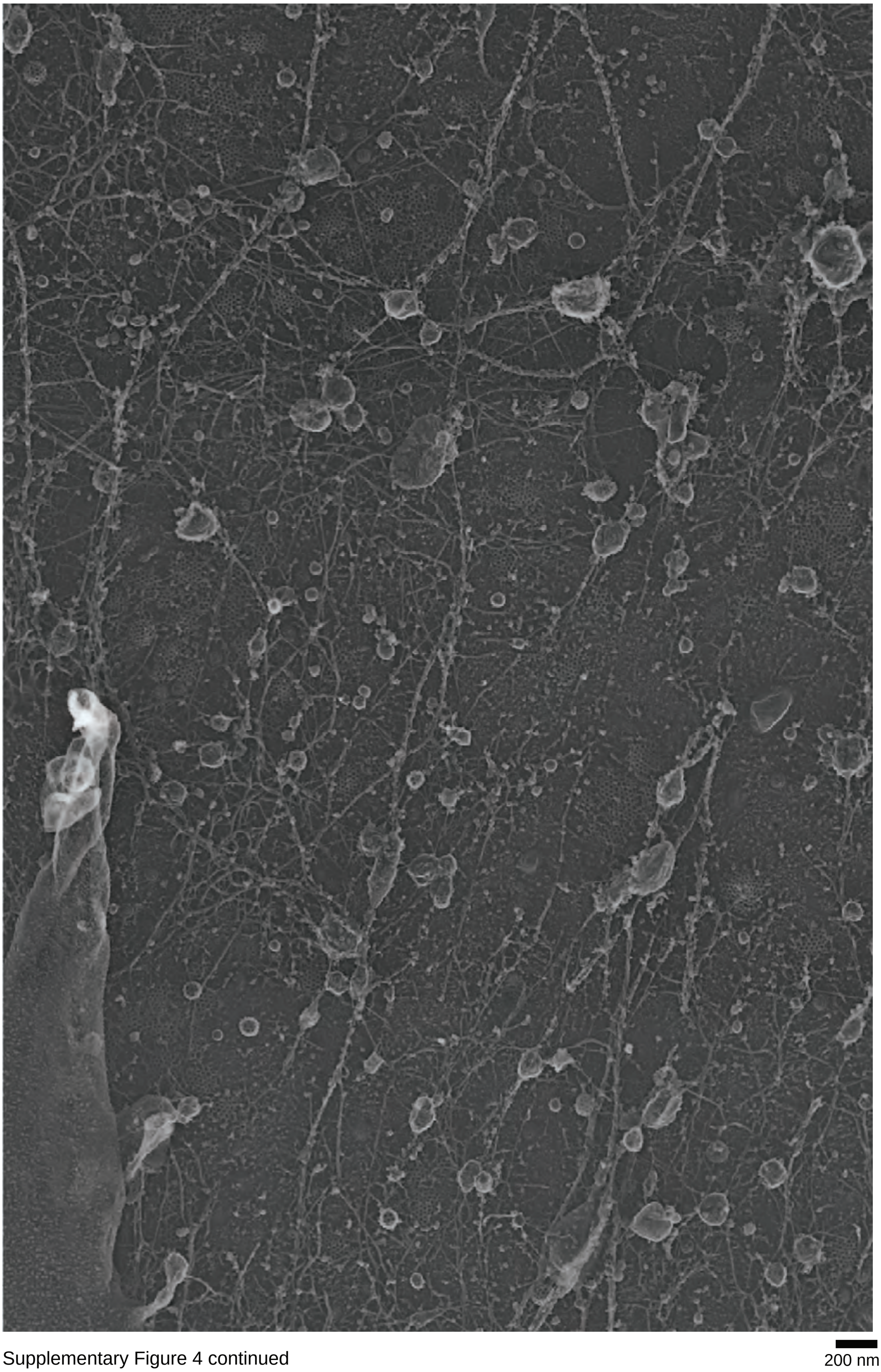

Supplementary Figure 4 continued

Gefi+EGF (Large image from crops in Fig. 2)

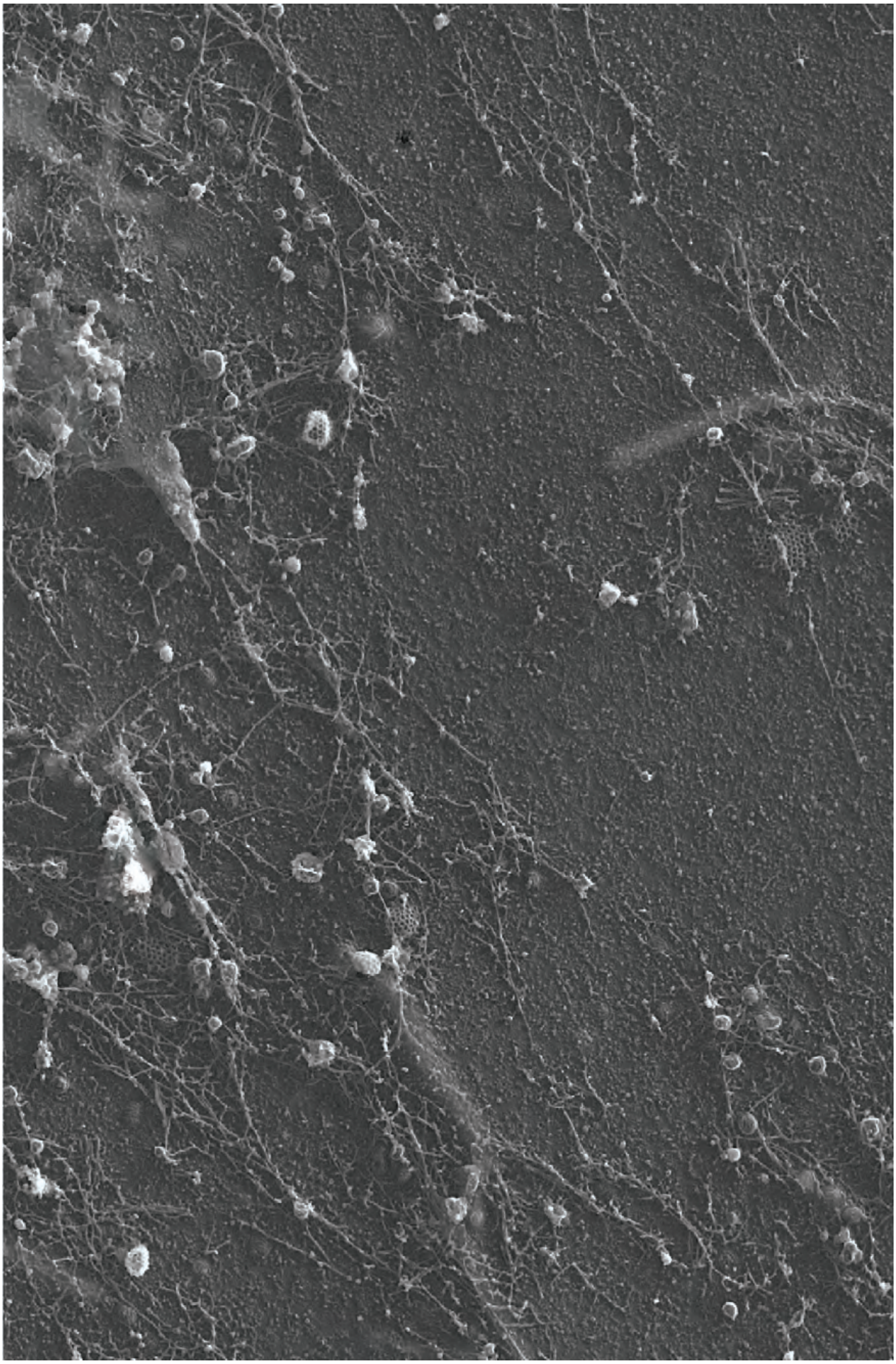

Supplementary Figure 4 continued

EGFR siRNA+EGF (Large image from crops in Fig. 2)

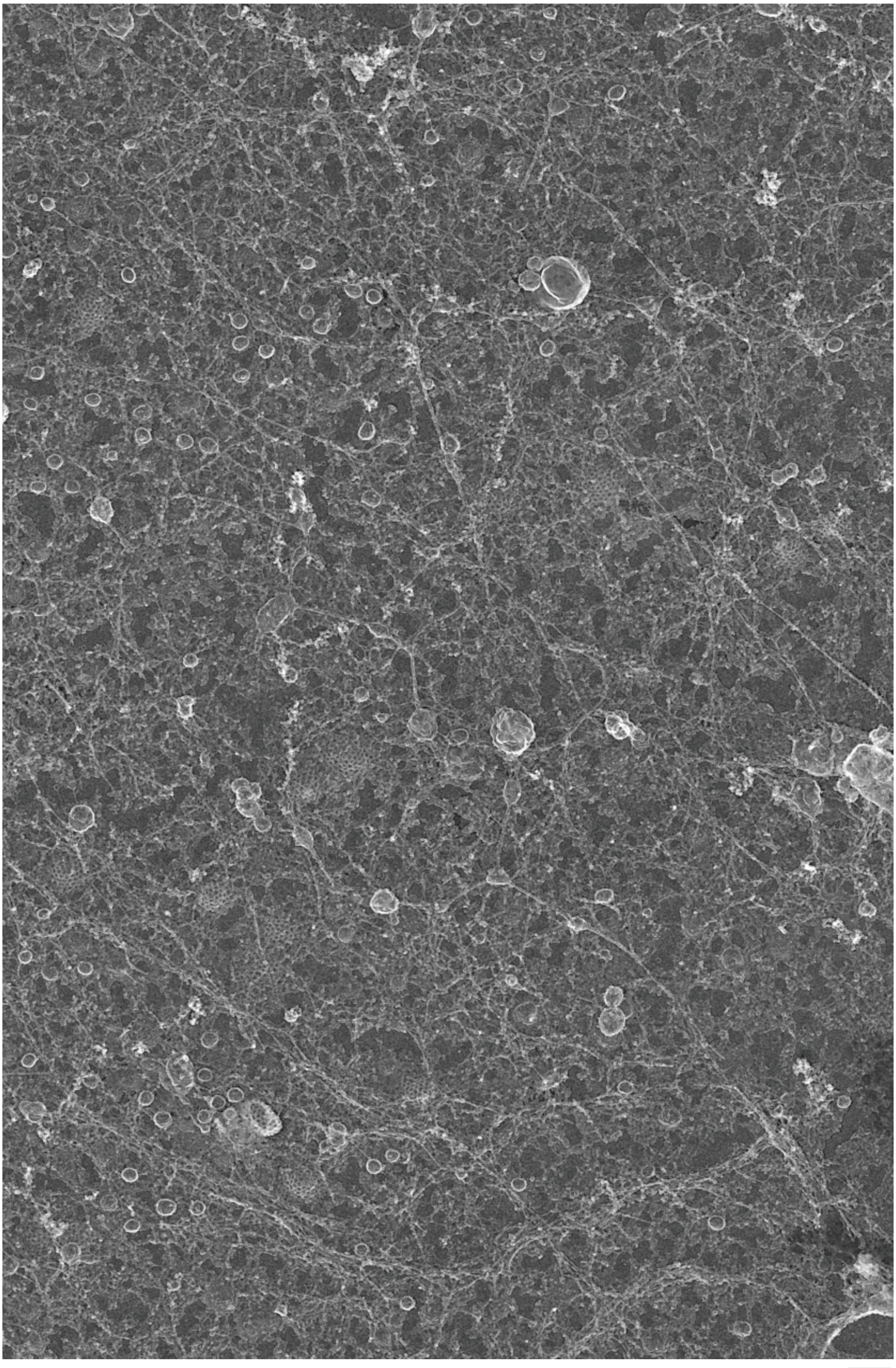

Supplementary Figure 4 continued

Ctrl (Large image from crops in Fig. 3)

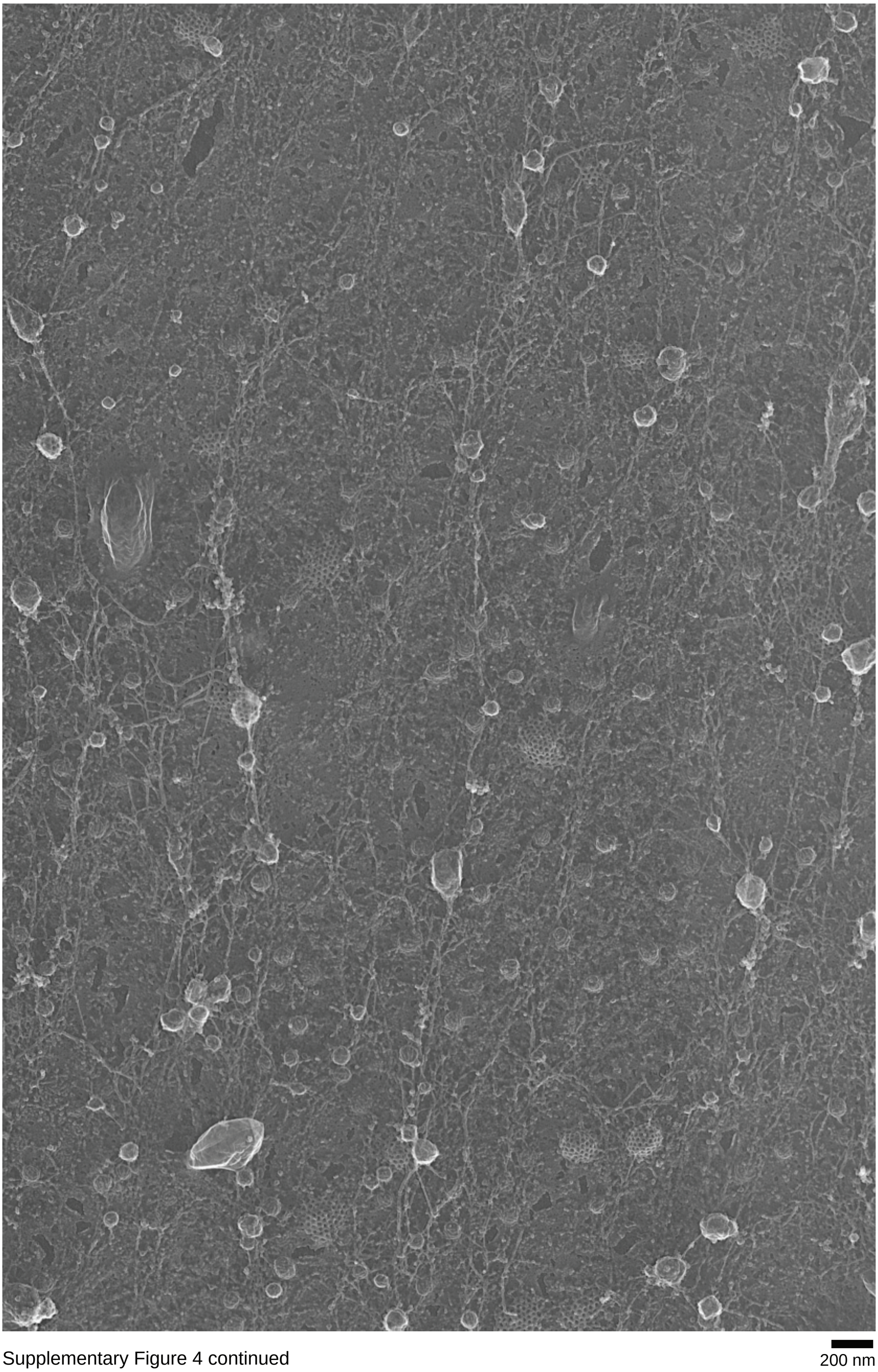

Supplementary Figure 4 continued

EGF (Large image from crops in Fig. 3)

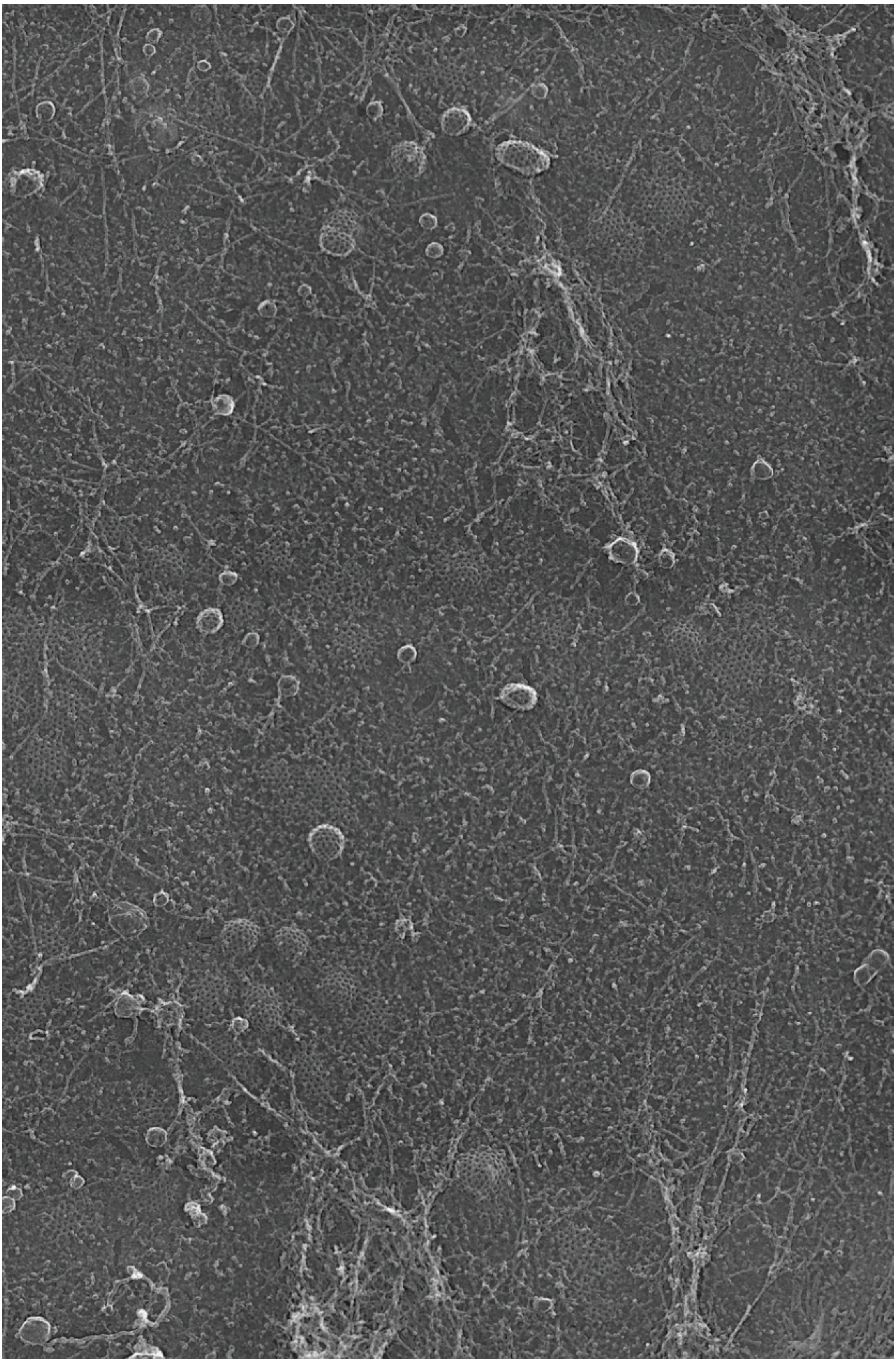

Supplementary Figure 4 continued

200 nm

PP2+EGF (Large image from crops in Fig. 3)

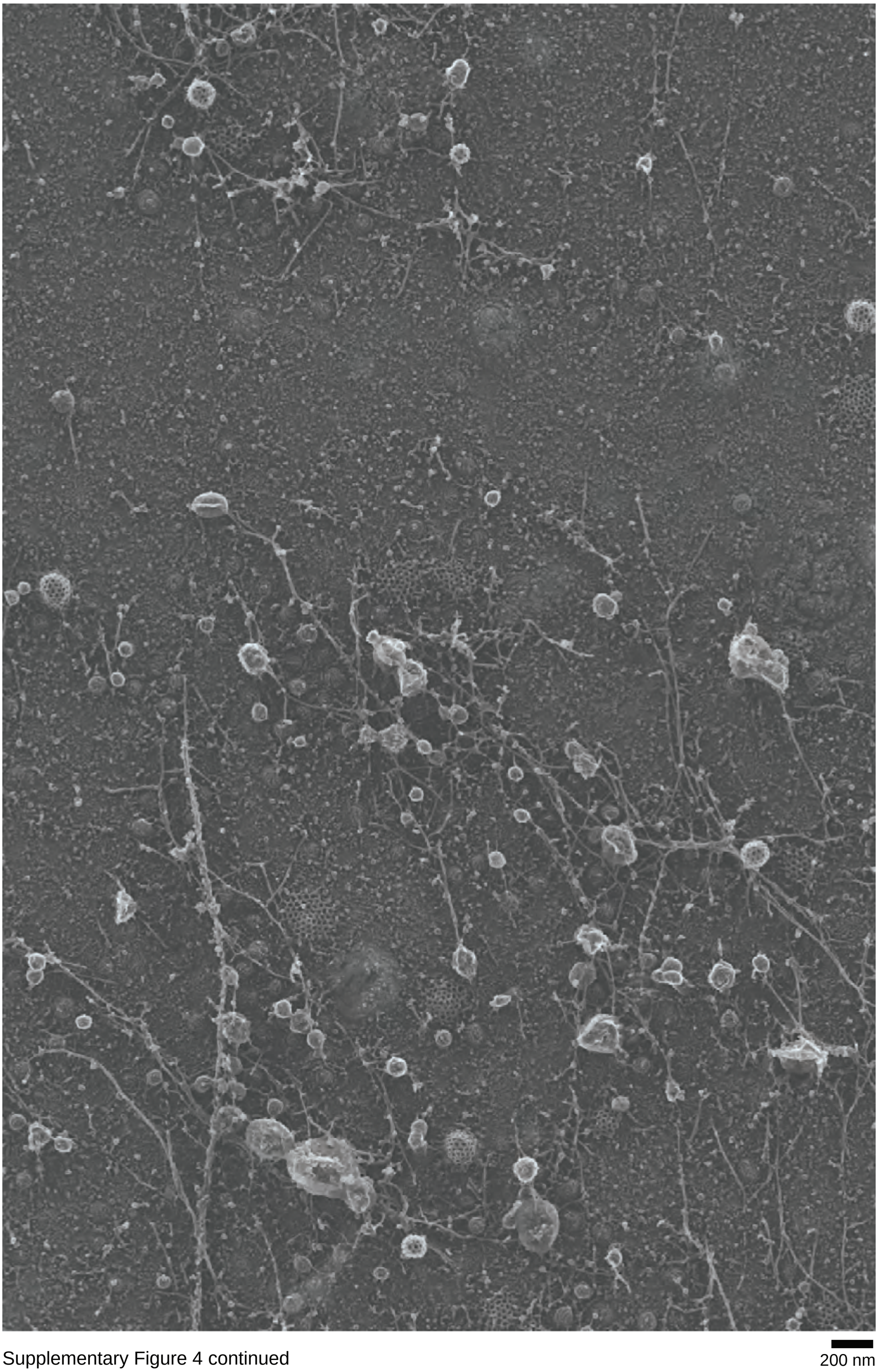

Supplementary Figure 4 continued

Src siRNA+EGF (Large image from crops in Fig. 3)

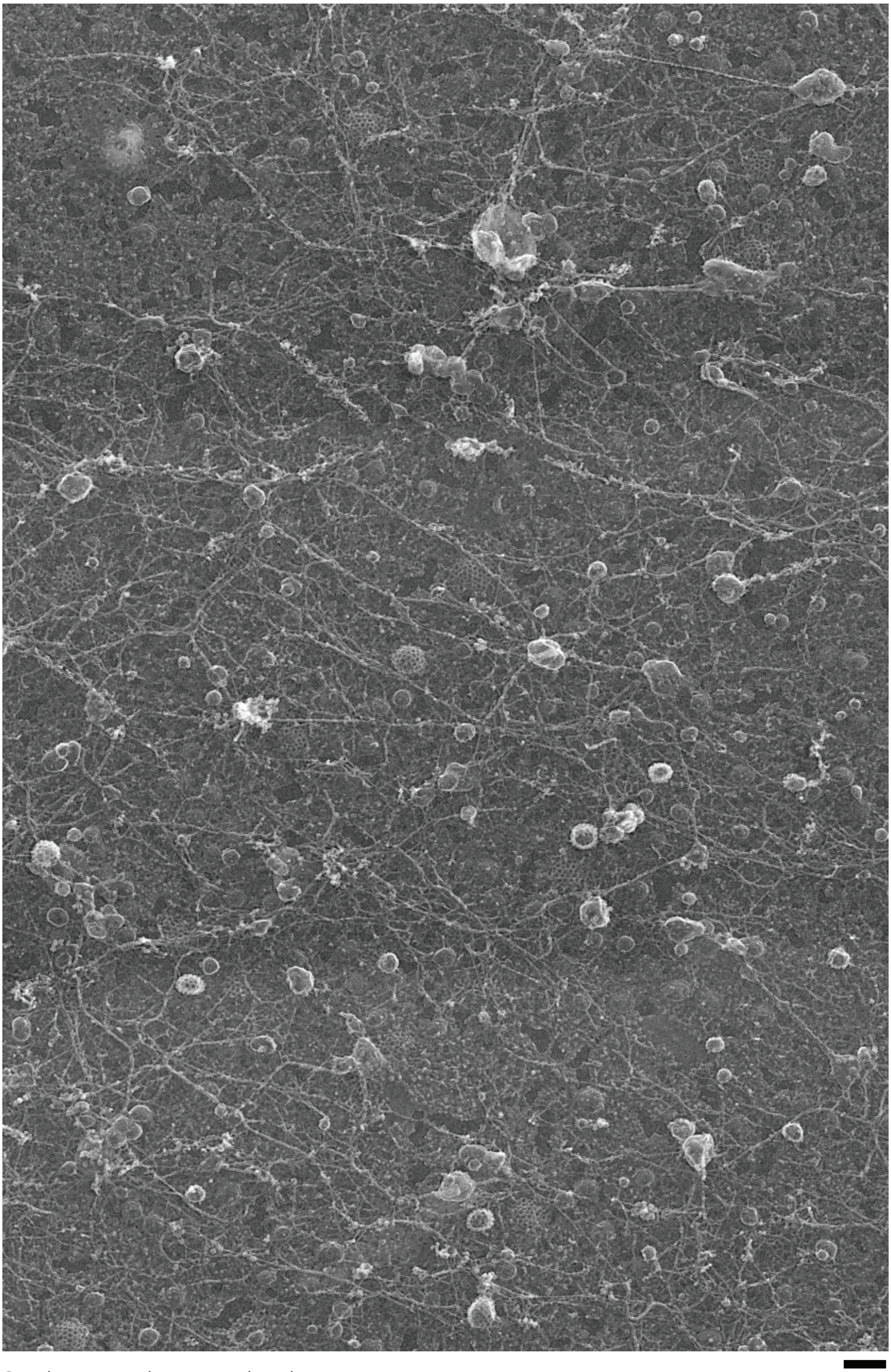

Supplementary Figure 4 continued

Ctrl (Large image from crops in Fig. 4)

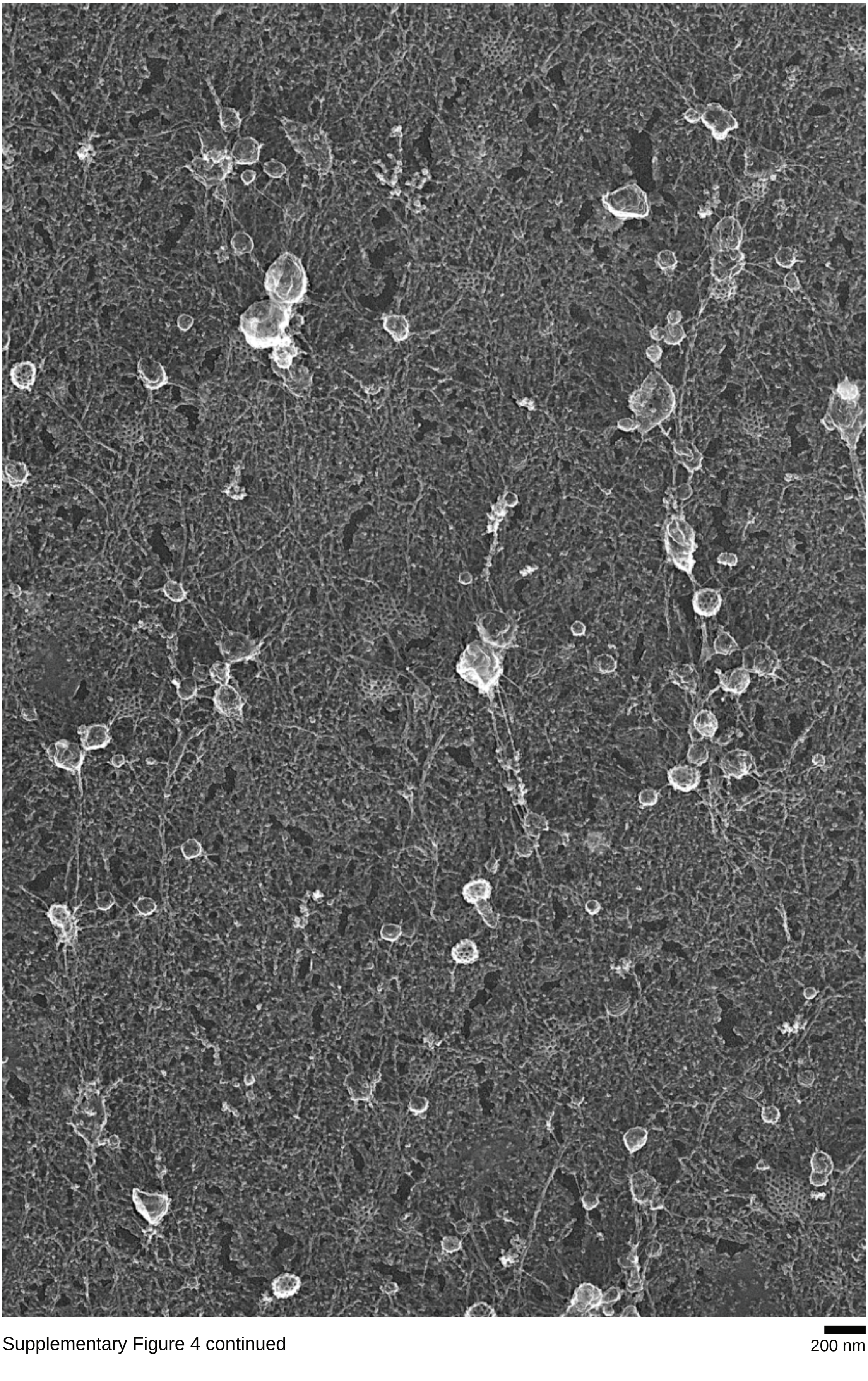

Supplementary Figure 4 continued

EGF (Large image from crops in Fig. 4)

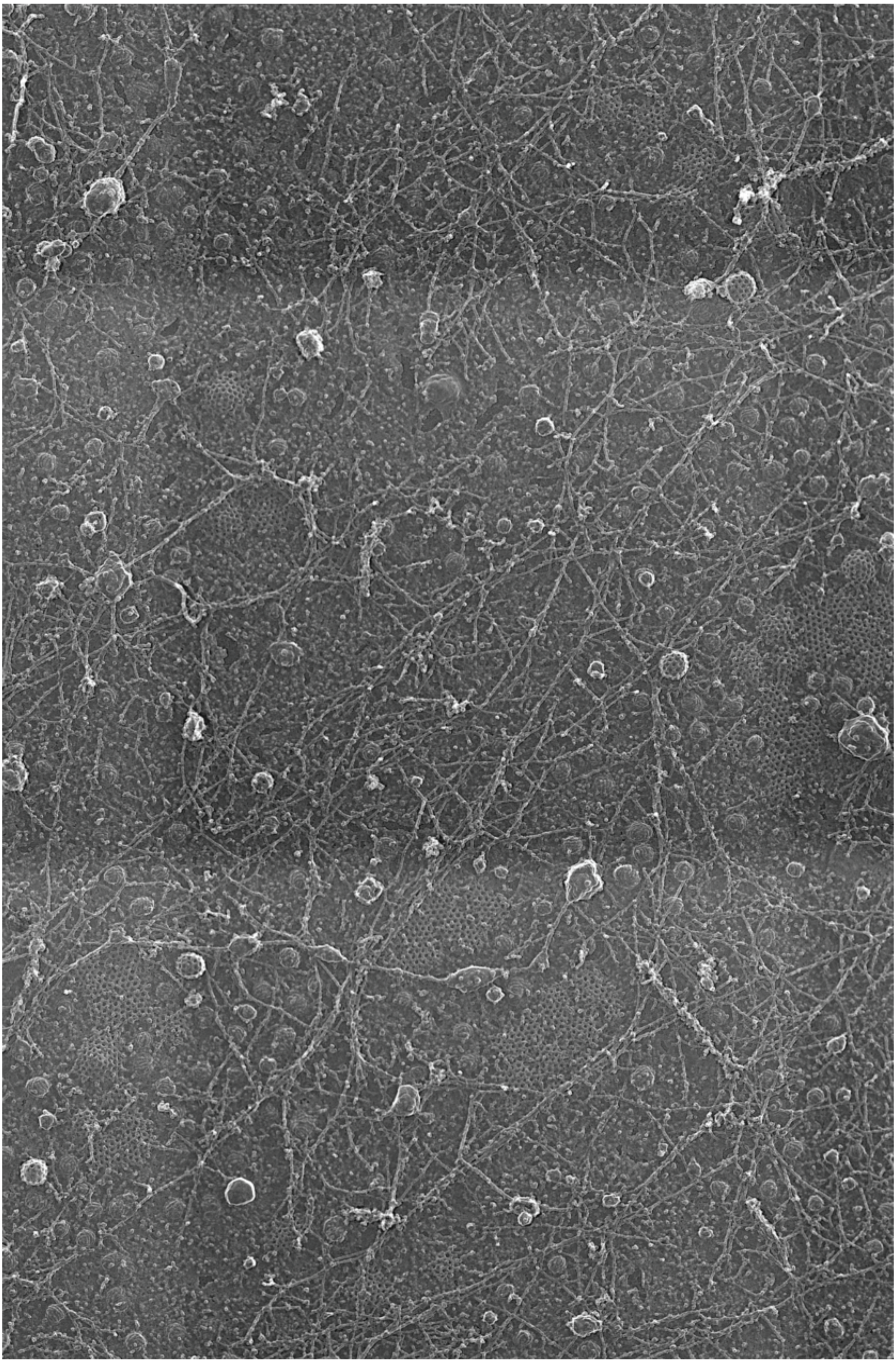

Supplementary Figure 4 continued

CTA+EGF (Large image from crops in Fig. 4)

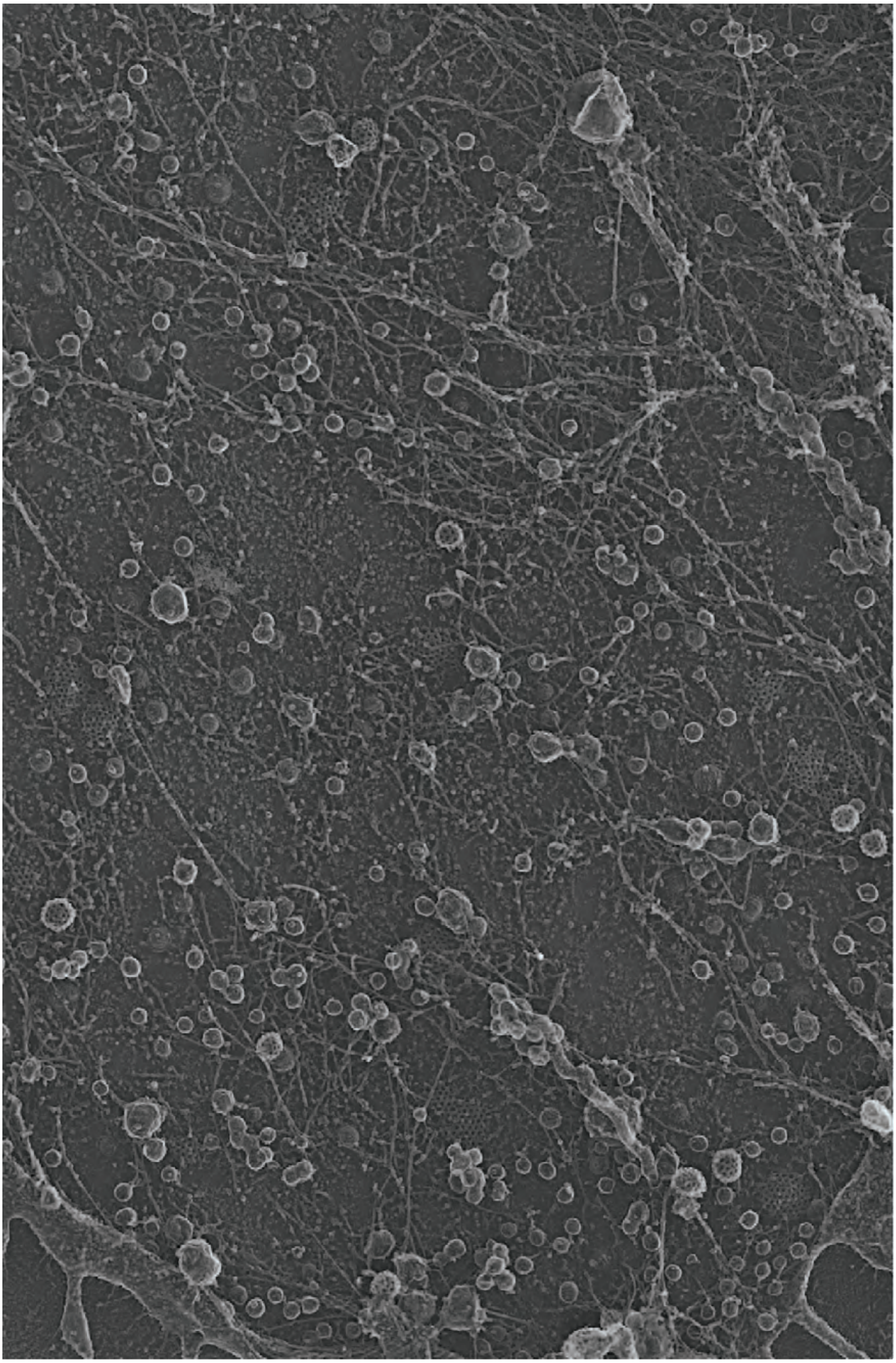

Supplementary Figure 4 continued

200 nm

β5-int siRNA+EGF (Large image from crops in Fig. 4)

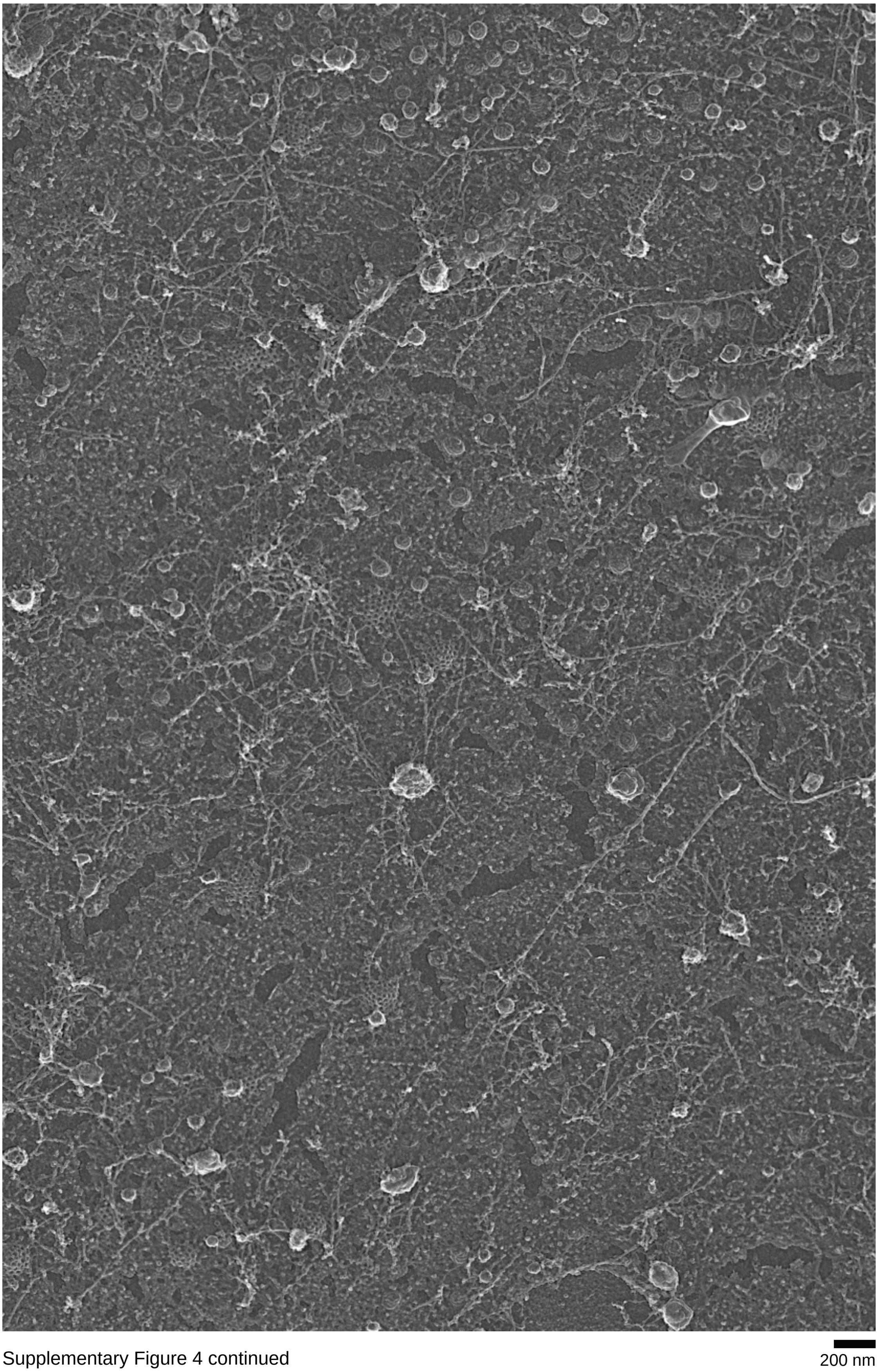

Supplementary Figure 4 continued

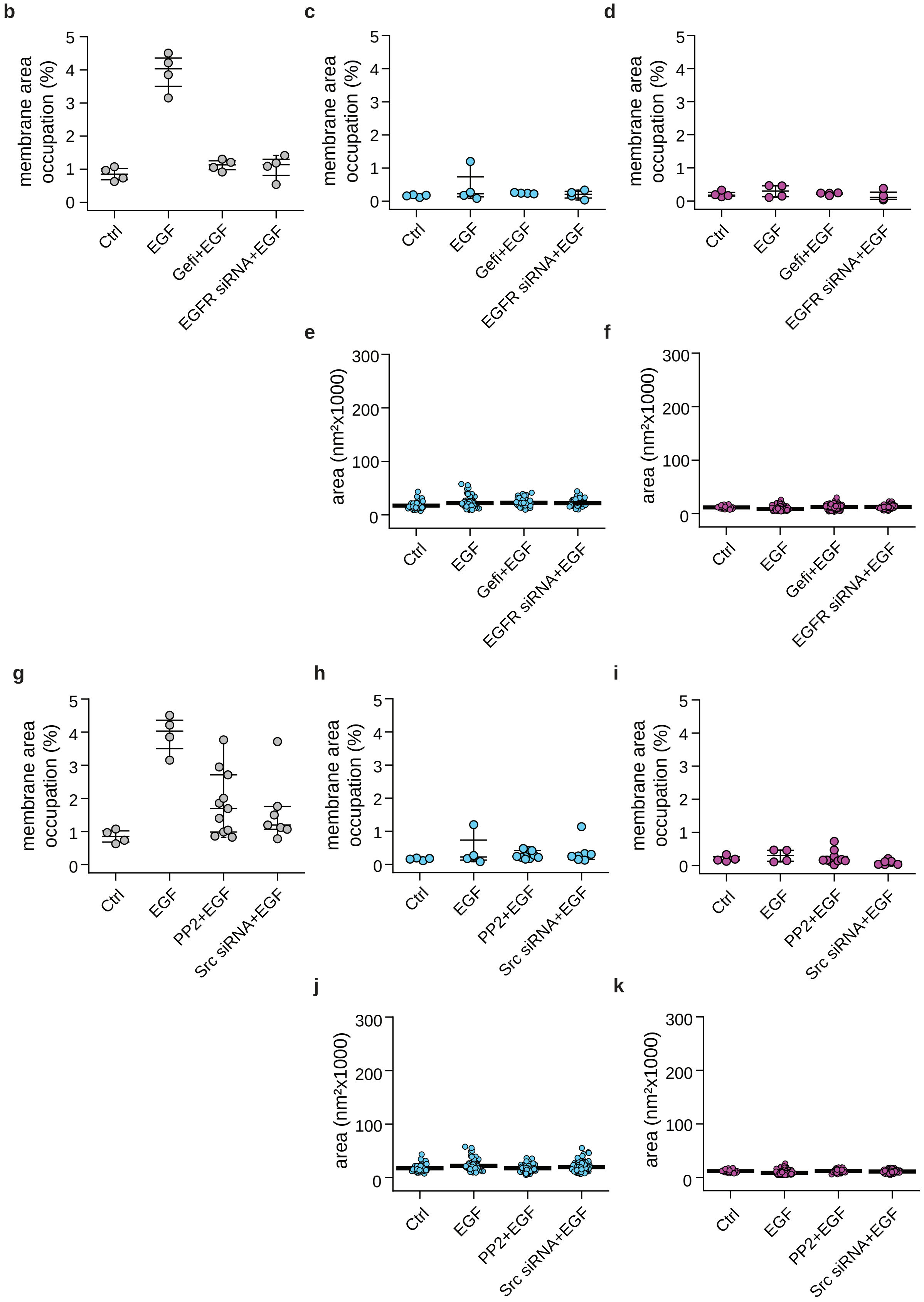

Supplementary Figure 4 continued

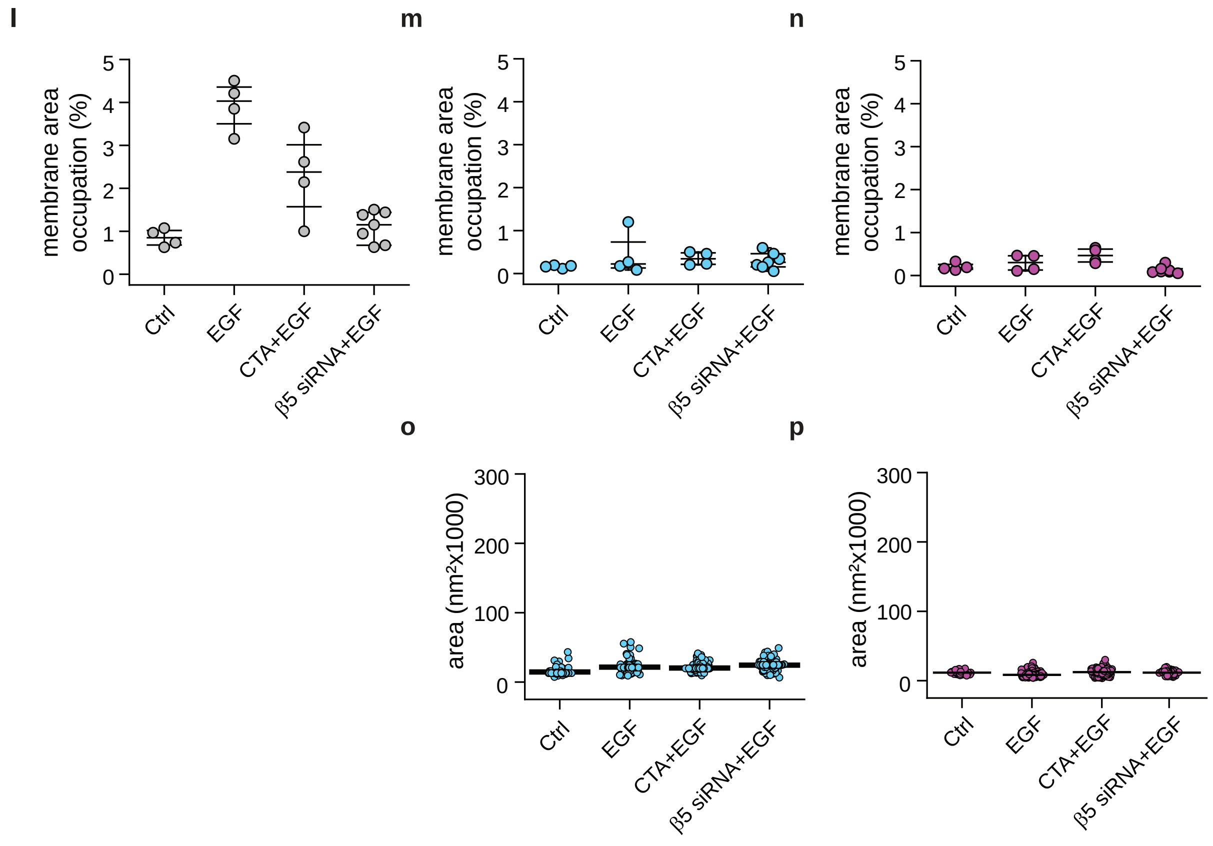

Supplementary Figure 4. Morphometric analysis of PREM images in Figures 2-4. a, Original PREM images of cells from which the cropped images and masks in Figures 2-4 were derived. Shown are control (Ctrl) HSC3-EGFR-GFP cells or treated with 50 ng/mL EGF for 15 min in the absence (EGF) or presence 10 μM gefitinib (Gefi+EGF), 30 pmol of EGFR siRNA (EGFR siRNA+EGF), 10 μM PP2 (PP2+EGF), 30 pmol of Src siRNA (Src siRNA+EGF), 10 μM cilengitide acid (CTA+EGF), and 120 pmol of β5-integrin siRNA (β5 siRNA+EGF). Scale bars are 200 nm. **b-n,** Morphometric analysis of the percentage of plasma membrane (PM) area occupation for (**b, g, l**) all CCSs, (**c, h, m**) dome, and (**d, i, n**) sphere structures in cells treated as in (**a**). I-shaped box plots show median extended from 25th to 75th percentiles, and minimum and maximum data point whiskers with a coefficient value of 1.5. **e-p,** Morphometric analysis of the size of (**e, j, p**) dome and (**f, k, p**) sphere clathrin structures in cells treated as in (**a**). Dot plots show every structure segmented, the bar indicate the median. N=2 biologically independent experiments with consistent results. Ctrl: N_{dome}=46, N_{sphere}=68; N_{cells}=4; EGF: N_{dome}=67, N_{sphere}=207, N_{cells}=4; Gefi+EGF: N_{dome}=68, N_{sphere}=167, N_{cells}=4; EGFR siRNA+EGF: N_{dome}=97, N_{sphere}=132; N_{cells}=4; PP2+EGF: N_{dome}=88, N_{sphere}=61, N_{cells}=11; Src siRNA+EGF: N_{dome}=286, N_{sphere}=145, N_{cells}=7; CTA+EGF: N_{dome}=68, N_{sphere}=167, N_{cells}=4; β5-siRNA+EGF: N_{dome}=107, N_{sphere}=83, N_{cells}=7. Ctrl and EGF data are from Figure 1 and shown for reference.

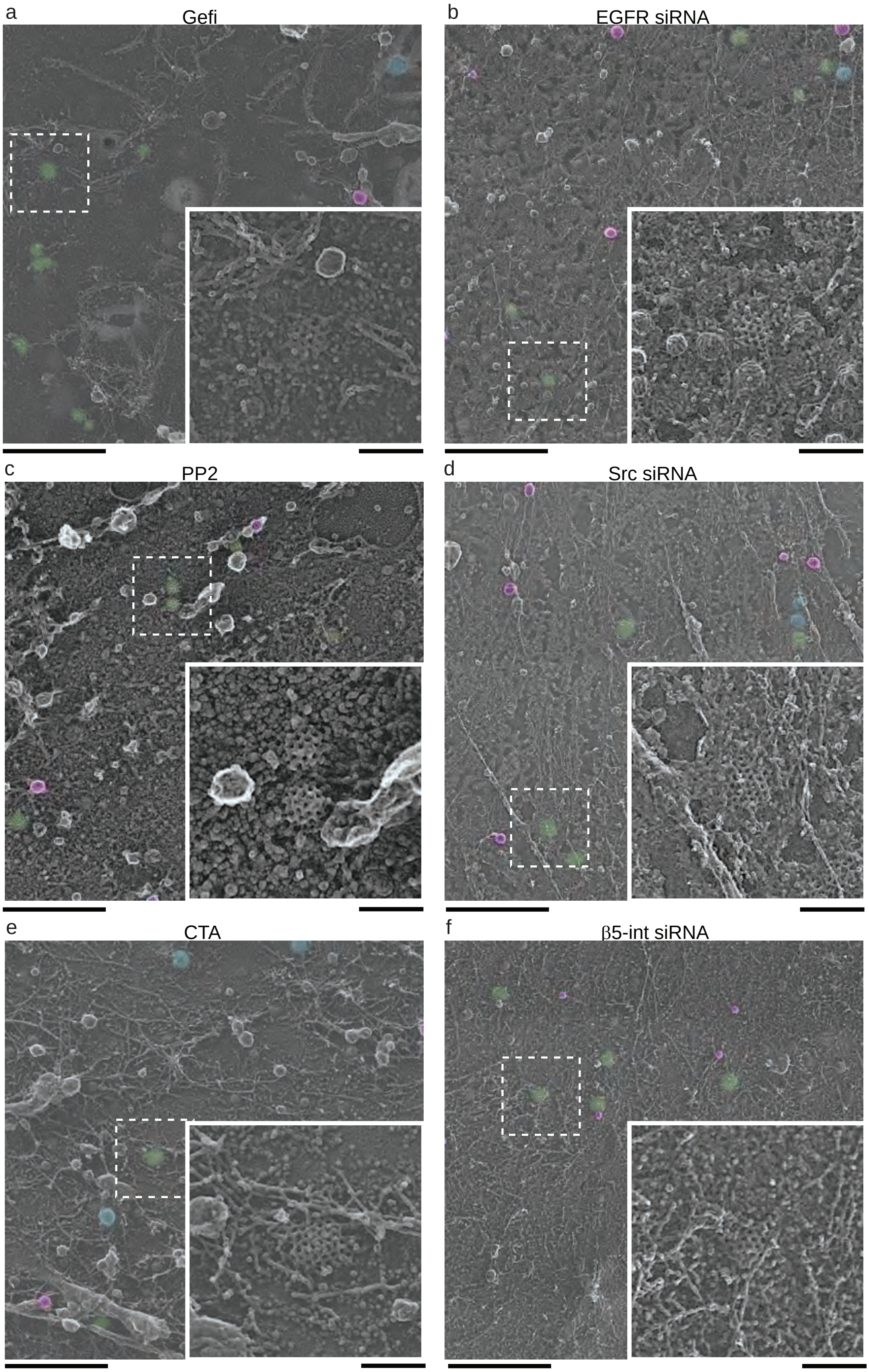

a
Gefi
b
EGFR siRNA
c
PP2
d
Src siRNA
e
CTA
f
β5-int siRNA

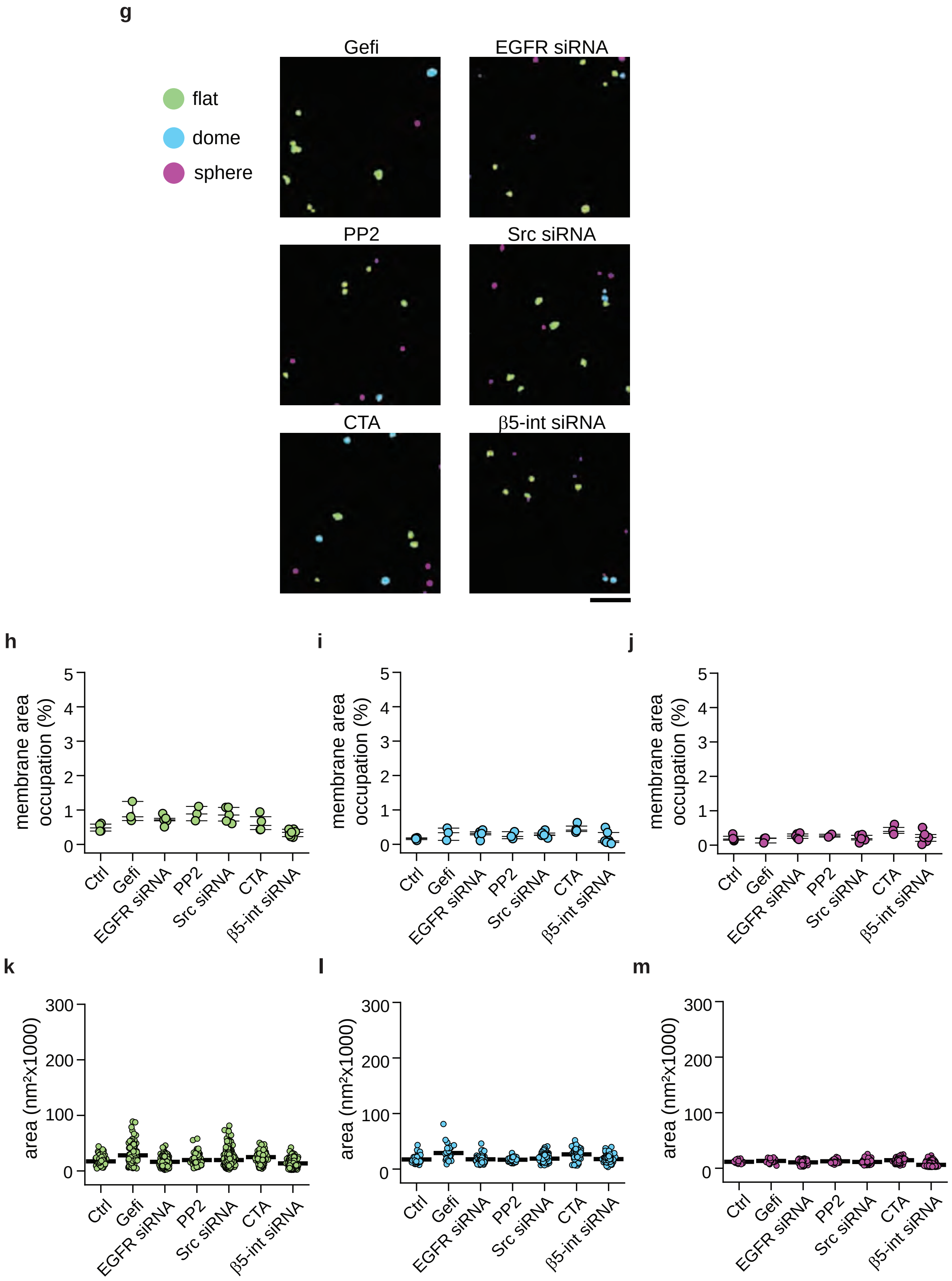

Supplementary Figure 5 continued

Supplementary Figure 5. Morphometric analysis of PREM images of cells treated with different drugs and siRNAs. a, Representative PREMs of control HSC3-EGFR-GFP cells treated with 10 μM gefitinib (Gefi), (**b**) 30 pmol of EGFR siRNA (EGFR siRNA), (**c**) 10 μM PP2 (PP2), (**d**) 30 pmol of Src siRNA (Src siRNA), (**e**) 10 μM cilengitide acid (CTA) or (**f**) 120 pmol of β5-integrin siRNA (β5- siRNA). The magnification insets are shown at the same scale and are outlined with dashed squares in each image. Flat, dome and sphere clathrin-coated structures (CCSs) are shown in green, blue and magenta, respectively, with native grayscale in magnified insets. **g,** Representative masks of segmented cells treated as in (**a-f**). **h-j,** Morphometric analysis of the percentage of plasma membrane (PM) area occupation for (**h**) flat, (**i**) dome, and (**j**) sphere CCSs. I-shaped box plots show median extended from 25th to 75th percentiles, and minimum and maximum data point whiskers with a coefficient value of 1.5. **k-m,** Morphometric analysis of the size of (**k**) flat, (**l**) dome, and (**j**) sphere CCSs of cells treated as indicated in (**a-f**). Dot plots show every structure segmented, the bar indicate the median. N=2 biologically independent experiments with consistent results. Ctrl: N_{flat}=141, N_{dome}=46, N_{sphere}=67; N_{cells}=4; Gefi: N_{flat}=118, N_{dome}=34, N_{sphere}=41, N_{cells}=3; EGFR siRNA: N_{flat}=308, N_{dome}=115, N_{sphere}=176, N_{cells}=5; PP2: N_{flat}=109, N_{dome}=36, N_{sphere}=53, N_{cells}=3; Src siRNA: N_{flat}=562, N_{dome}=216, N_{sphere}=226, N_{cells}=5; CTA: N_{flat}=171, N_{dome}=137, N_{sphere}=229, N_{cells}=4; β5-siRNA: N_{flat}=293, N_{dome}=180, N_{sphere}=637, N_{cells}=8. Scale bars in (**a-g**) are 1 μm; insets are 200 nm. Ctrl data are from Figure 1 and shown for reference.

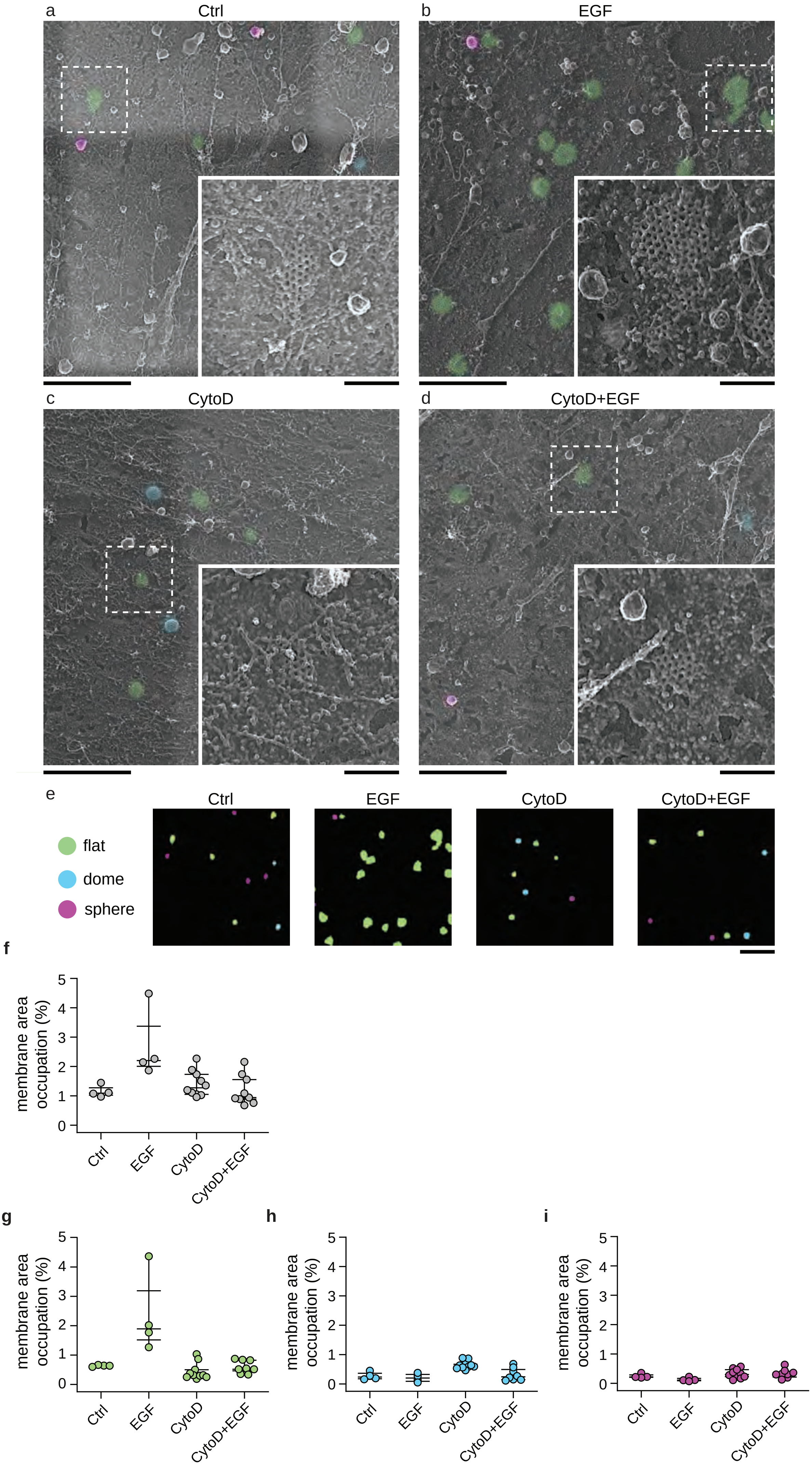
a
Ctrl
b
EGF
c
CytoD
d
CytoD+EGF
e
Ctrl
EGF
CytoD
CytoD+EGF
flat
dome
sphere
f
membrane area occupation (%)
5
4
3
2
1
0
Ctrl
EGF
CytoD
CytoD+EGF
g
h
i

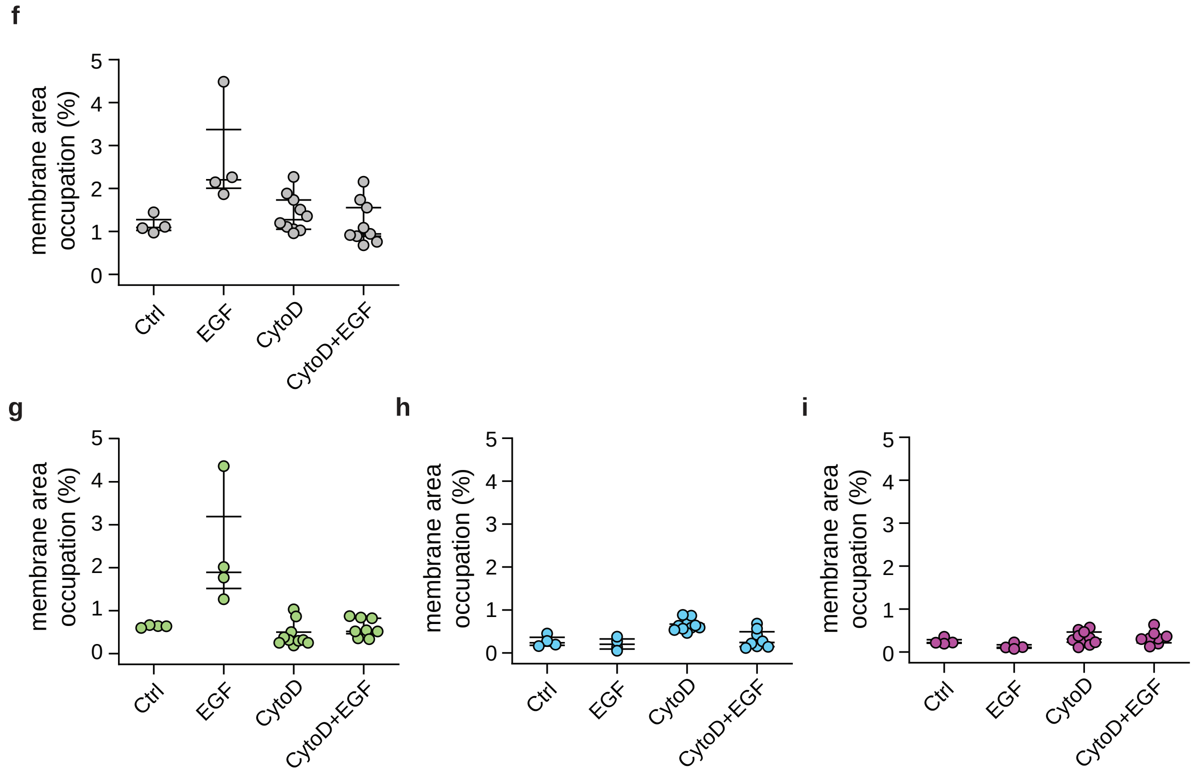

Supplementary Figure 6. Role of actin cytoskeleton in flat clathrin lattice expansion. a, Representative PREMs of control HSC3-EGFR-GFP cells (Ctrl), (**b**) cells treated either with 50 ng/mL EGF (EGF) or (**c**) 10 μM of the actin polymerization inhibitor cytochalasin D alone for 15 min (CytoD), (**d**) and both (CytoD+EGF). The magnification insets are shown at the same scale and are outlined with dashed squares in each image. Flat, dome and sphere clathrin-coated structures (CCSs) are shown in green, blue and magenta, respectively, with native grayscale in magnified insets. **e,** Representative masks of segmented cells treated as in (**a-d**). **f-i,** Morphometric analysis of the percentage of plasma membrane (PM) area occupation for (**f**) all CCSs, (**g**) flat, (**h**) dome, and (**i**) sphere CCSs in cells treated as in (**a-d**). I-shaped box plots show median extended from 25th to 75th percentiles, and minimum and maximum data point whiskers with a coefficient value of 1.5. **j-l,** Morphometric analysis of the size of (**j**) flat, (**k**) dome and (**l**) sphere CCSs in cells treated as in (**a-d**). Dot plots show every structure segmented, the bar indicate the median. N=2 biologically independent experiments in (**f-l**) with consistent results; Ctrl: N_{flat}=335, N_{dome}=115, N_{sphere}=164; N_{cells}=4; EGF: N_{flat}=423, N_{dome}=63, N_{sphere}=99; N_{cells}=4; CytoD: N_{flat}=236, N_{dome}=422, N_{sphere}=360; N_{cells}=10; CytoD+EGF: N_{flat}=238, N_{dome}=155, N_{sphere}=319; N_{cells}=9. Scale bars in (**a-e**) are 1 μm; insets are 200 nm. Ctrl and EGF data are from Supplementary Figure 6 and shown for reference. **m,** Automated correlation analysis of TIRF images of HSC3 WT cells co-transfected with mScarlet-CLCa and F-tractin-GFP and treated as in (**a-d**). Dot box plots show median extended from 25th to 75th percentiles, mean (square) and minimum and maximum data point whiskers with a coefficient value of 1.5. N=3 independent experiments wih consistent results. N_{Ctrl}= 19 cells– 954 spots; N_{EGF}= 19 cells– 1141 spots; N_{CytoD}= 20 cells– 983 spots; $N_{CytoD+GF}$= 19 cells– 1198 spots.

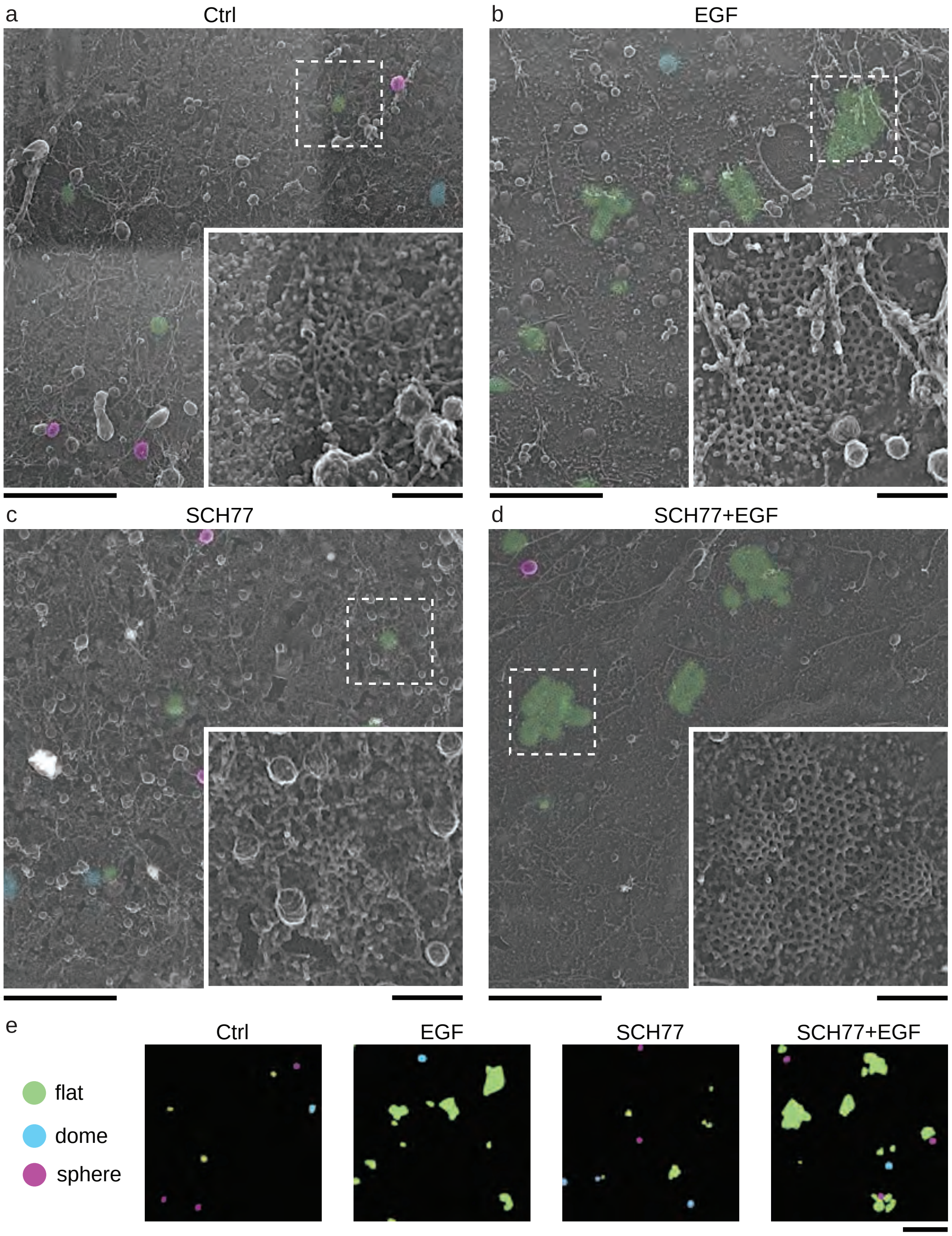
a
Ctrl
b
EGF
c
SCH77
d
SCH77+EGF
e
Ctrl
EGF
SCH77
SCH77+EGF
flat
dome
sphere

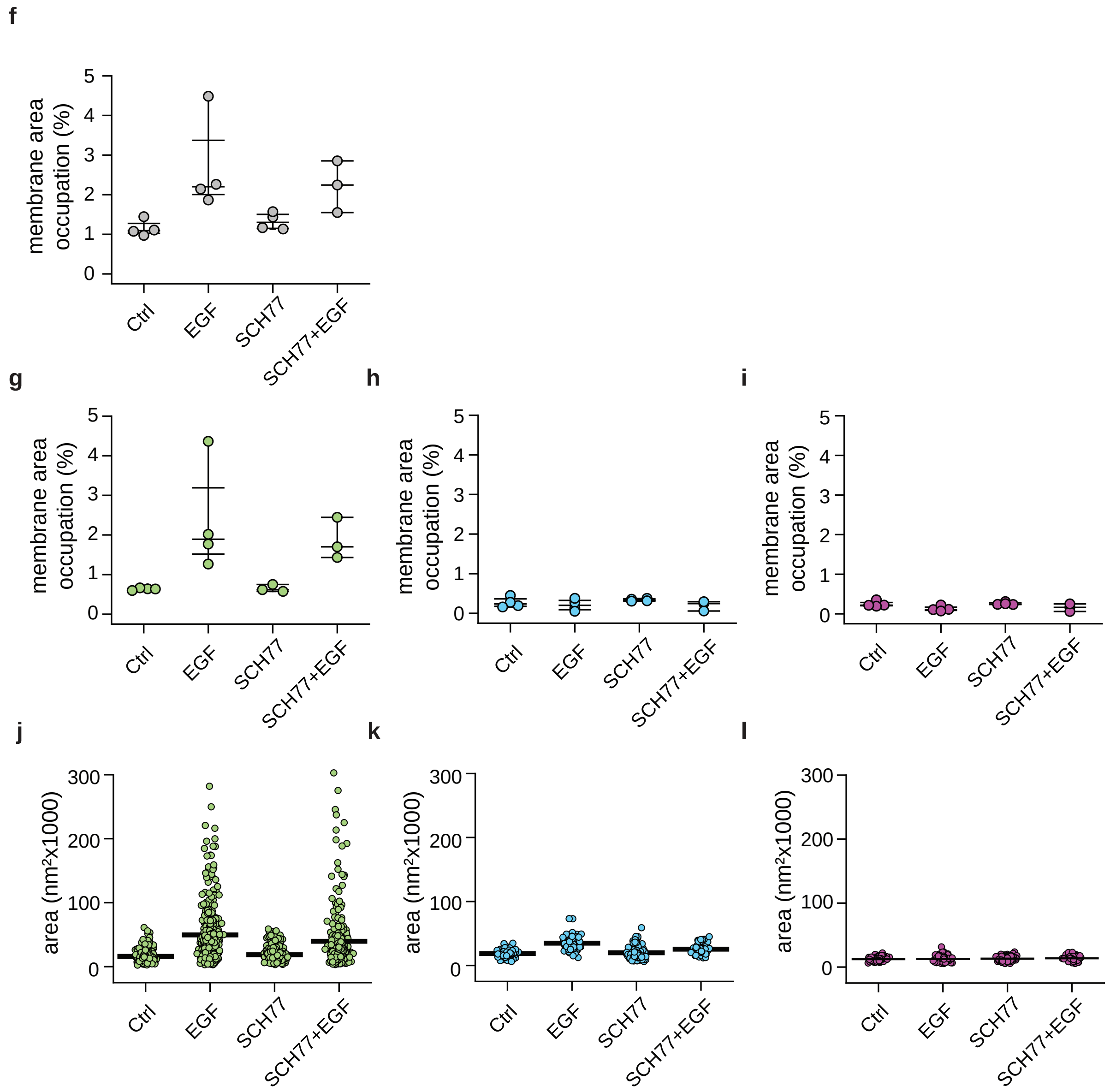

Supplementary Figure 7. Flat clathrin lattice expansion is ERK independent. a, Representative PREMs of control HSC3-EGFR-GFP cells (Ctrl), (**b**) treated either with 50 ng/mL EGF (EGF) or (**c**) 10 µM of the ERK inhibitor SCH772984 alone for 15 min (SCH77), (**d**) and both (SCH77+EGF). The magnification insets are shown at the same scale and are outlined with dashed squares in each image. Flat, dome and sphere clathrin-coated structures (CCSs) are shown in green, blue and magenta, respectively, with native grayscale in magnified insets. **e,** Representative masks of segmented cells treated as in (**a-d**). **f-i,** Morphometric analysis of the percentage of plasma membrane (PM) area occupation for (**f**) all CCSs, (**g**) flat, (**h**) dome, and (**i**) sphere CCSs in cells treated as in (**a-d**). I-shaped box plots show median extended from 25th to 75th percentiles, and minimum and maximum data point whiskers with a coefficient value of 1.5. **j-l,** Morphometric analysis of the size of (**j**) flat, (**k**) dome and (**l**) sphere CCSs in cells treated as in (**a-d**). Dot plots show every structure segmented; the bar indicate the median. Ctrl: N_{flat}=335, N_{dome}=115, N_{sphere}=164; N_{cells}=4; EGF: N_{flat}=423, N_{dome}=63, N_{sphere}=99; N_{cells}=4; SCH77: N_{flat}=441, N_{dome}=197, N_{sphere}=228; N_{cells}=4; SCH77+EGF: N_{flat}=316, N_{dome}=61, N_{sphere}=94; N_{cells}=3. Number of independent experiments=2 (**f-m**). Scale bars in (**a-e**) are 1 µm; insets are 200 nm.

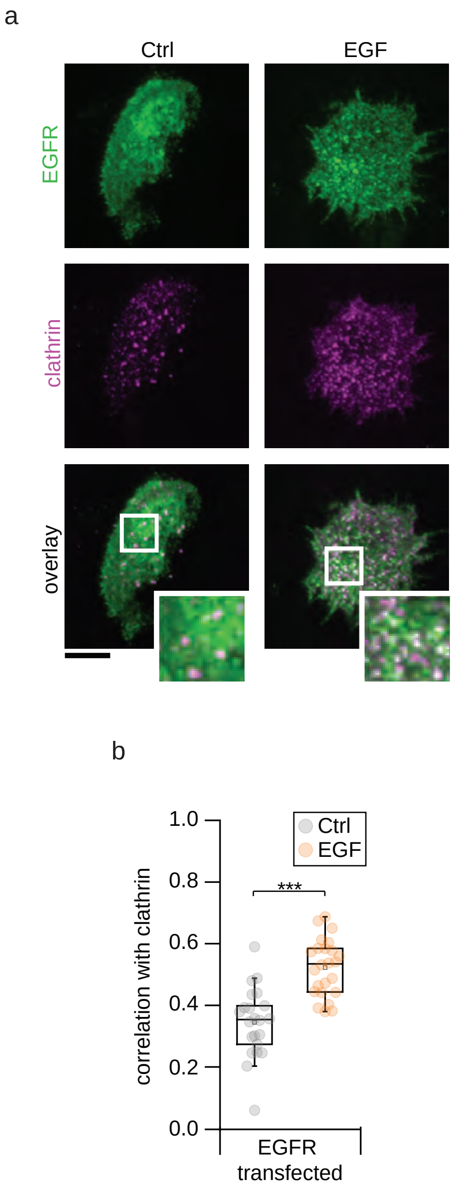

Supplementary Figure 8. Over-expressed EGFR-GFP correlates with clathrin after EGF stimulation. a, Representative TIRF images of HSC3 WT cells co-transfected with mScarlet-CLCa and EGFR-GFP before (Ctrl) or after 50 ng/mL EGF stimulation for 15 min. **b,** Automated correlation analysis of (**a**). Significance was tested by a two-tailed *t*-test, $*P_{EGFR}$=4.2×10^{-7}. $N_{EGFR\text{-}Ctrl}$= 22 cells – 1117 spots, $N_{EGFR\text{-}EGF}$=24 cells – 1305 spots. Number of independent experiments=4. Dot box plots show median extended from 25th to 75th percentiles, mean (square) and minimum and maximum data point whiskers with a coefficient value of 1.5. Scale bar is 10 μm; insets are 7.3x7.3 μm.

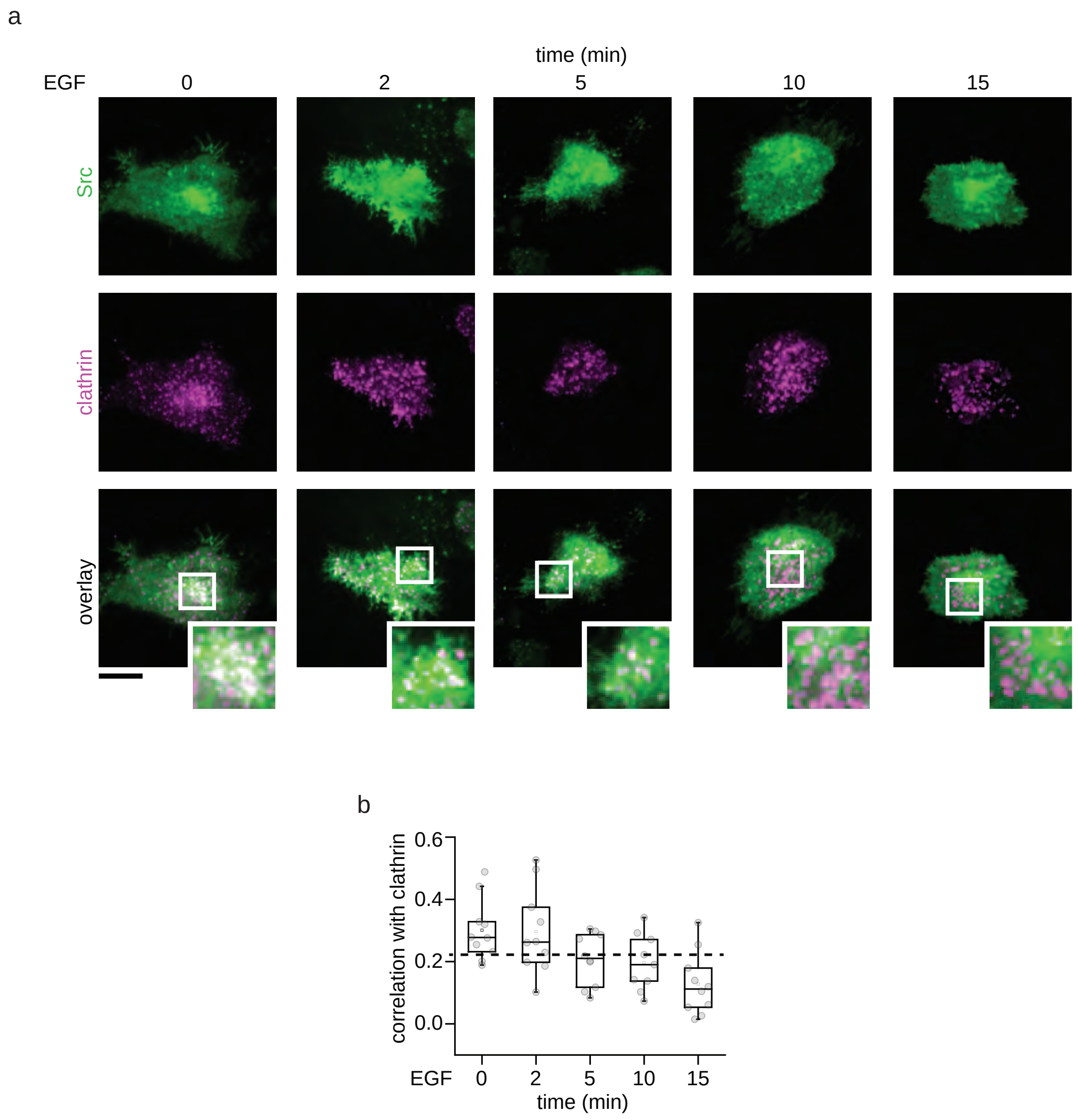

Supplementary Figure 9. Time course of Src correlation with clathrin after EGF stimulation. a, Representative TIRF images of HSC3 WT cells co-transfected with Src-GFP and mScarlet-CLCa before or after 50 ng/mL EGF stimulation for 2, 5, 10 and 15 min. **b,** Automated correlation analysis of (**a**). N=2 biologically independent experiments with consistent results. $N_{0\ min}$=10 cells – 933 spots; $N_{2\ min}$=10 cells – 850 spots; $N_{5\ min}$=10 cells – 1223 spots; $N_{10\ min}$=9 cells – 1050 spots; $N_{15\ min}$=10 cells – 1128 spots examined over the indicated independent experiments. Dot box plots show median extended from 25th to 75th percentiles, mean (square) and minimum and maximum data point whiskers with a coefficient value of 1.5. Scale bar is 10 μm; insets are 7.3x7.3 μm.

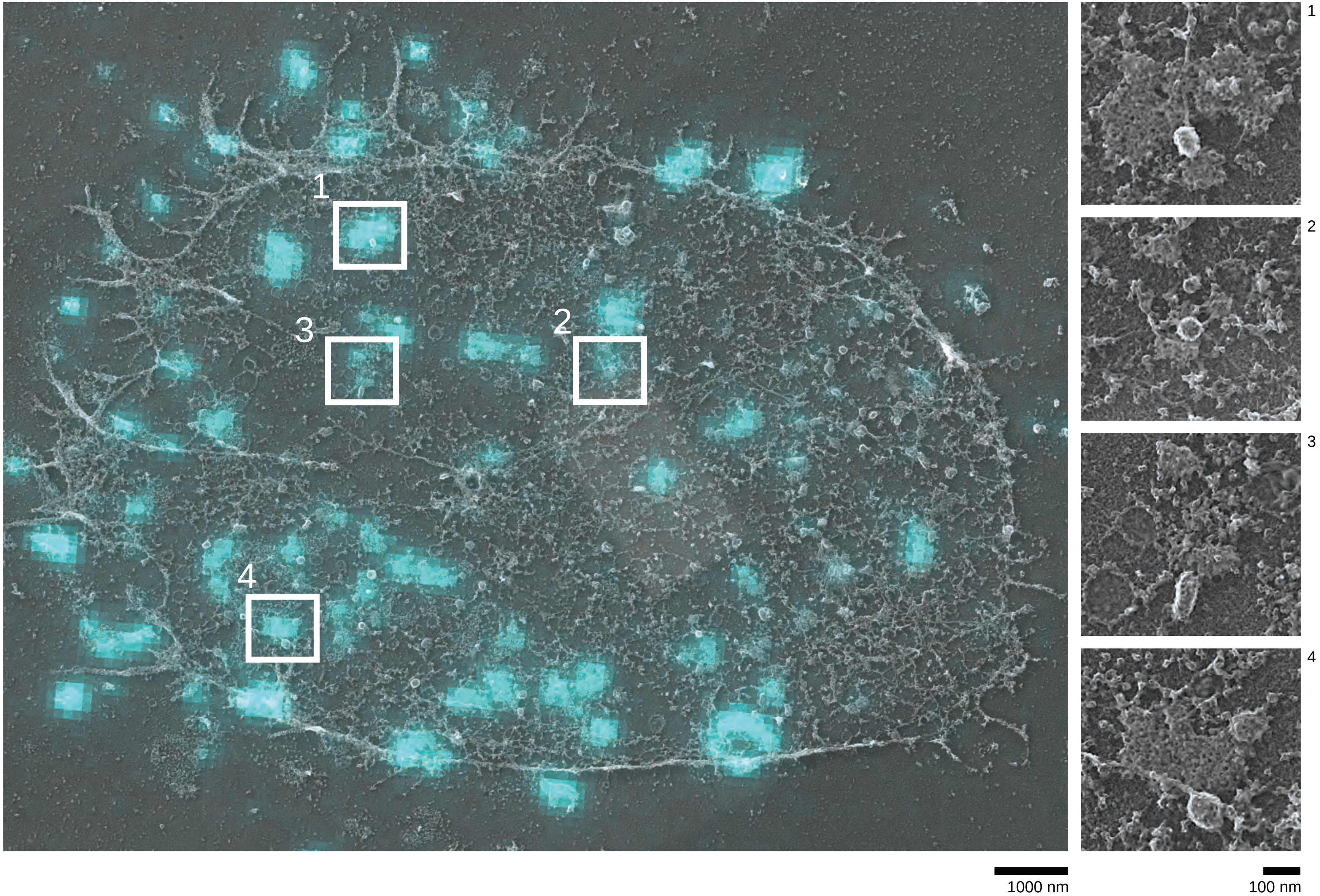

Supplementary Figure 10. Correlative TIRF and electron microscopy of β5-integrin. TIRF β5-integrin-GFP image (cyan) overlaid with PREM image (gray) of the unroofed HSC3 WT cell membrane. Fluorescence from β5-integrin-GFP overlaps with flat, domed, and spherical clathrin coated structures (right panel) from white boxes in the left panel indicated with numbers. N=2 biologically independent experiments with consistent results. Scale bars are 1000 nm and 100 nm for left and right panels, respectively.

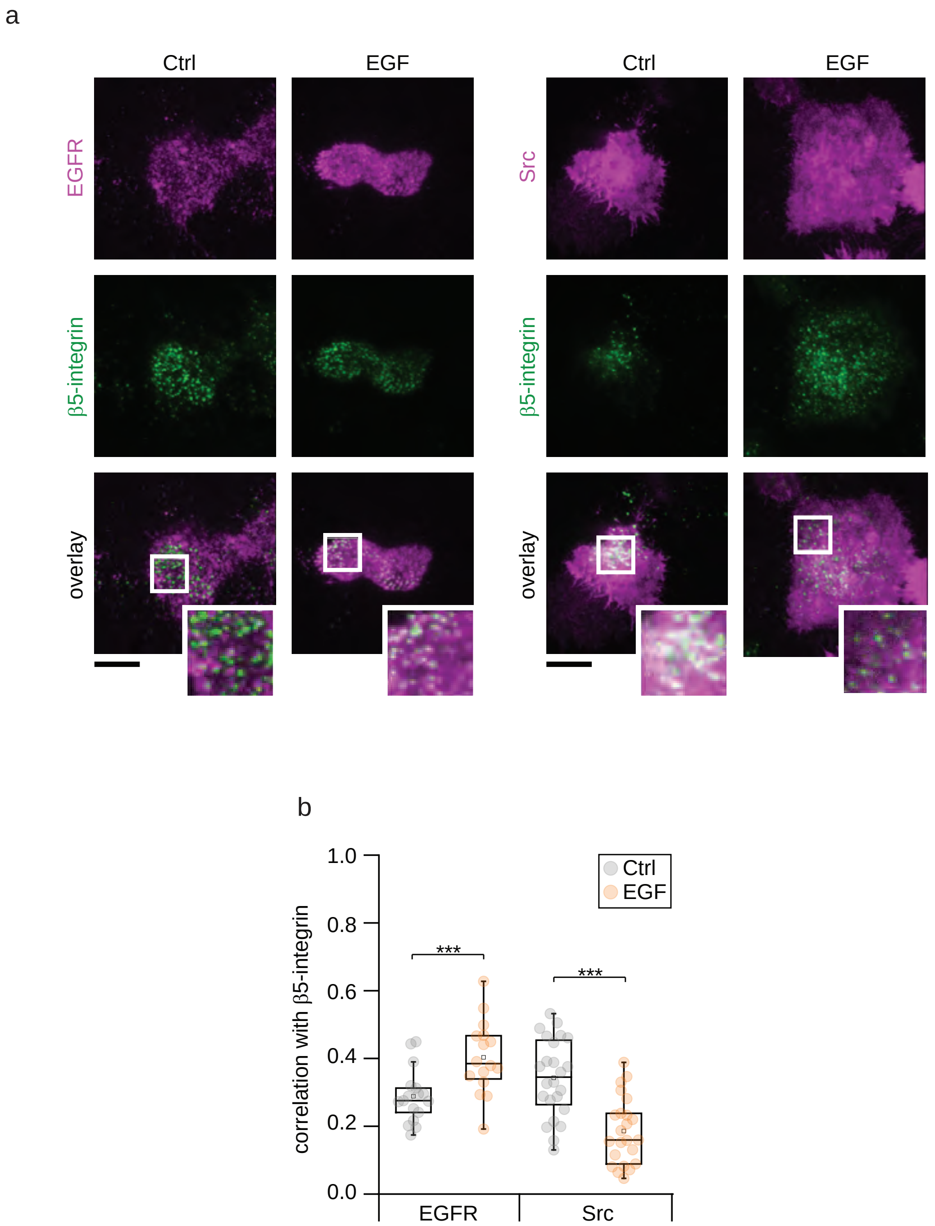

Supplementary Figure 11. Differential location of EGFR and Src in β5-integrin enriched structures. a, Representative TIRF images of HSC3 WT cells co-transfected with β5-integrin-GFP and either EGFR-mScarlet or mCherry-Src before (Ctrl) or after 50 ng/mL EGF stimulation for 15 min. **b,** Automated correlation analysis of (**a**). Significance was tested by a two-tailed *t*-test $*P_{EGFR} = 8.4\times10^{-4}$, $*P_{Src} = 1.2\times10^{-6}$. N=3 biologically independent experiments with consistent results. $N_{EGFR\text{-}Ctrl}$=17 cells – 1516 spots, $N_{EGFR\text{-}EGF}$=16 cells – 1416 spots, $N_{Src\text{-}Ctrl}$= 24 cells – 1936 spots, $N_{Src\text{-}EGF}$=23 cells – 1872 spots examined over the indicated independent experiments. Dot box plots show median extended from 25th to 75th percentiles, mean (square) and minimum and maximum data point whiskers with a coefficient value of 1.5. Scale bar is 10 μm; insets are 7.3x7.3 μm.

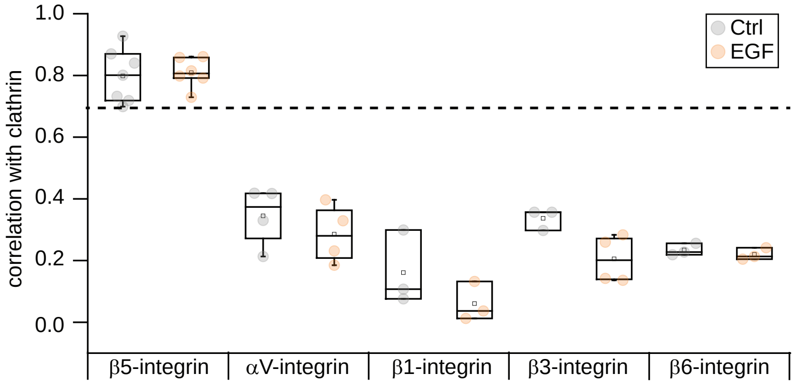

Supplementary Figure 12. Correlation of clathrin-coated structures with different integrins. Automated correlation analysis of HSC3 WT cells co-transfected with mScarlet-CLCa and the indicated integrin tagged with GFP before (Ctrl) or after 50 ng/mL EGF stimulation for 15 min. Cell were imaged using TIRFM. Dot box plots show median extended from 25th to 75th percentiles, mean (square) and minimum and maximum data point whiskers with a coefficient value of 1.5. Dots represent the mean correlation value of all the spots analyzed from independent experiments. $N_{\beta5\text{-Ctrl}}$=7, $N_{\beta5\text{-EGF}}$=6, $N_{\alpha V\text{-Ctrl}}$=4, $N_{\alpha V\text{-EGF}}$=4, $N_{\beta1\text{-Ctrl}}$=3, $N_{\beta5\text{-EGF}}$=3, $N_{\beta3\text{-Ctrl}}$=3, $N_{\beta3\text{-EGF}}$=4, $N_{\beta6\text{-Ctrl}}$=3, $N_{\beta5\text{-EGF}}$=3 biologically independent experiments with consistent results, respectively.

a

β5-integrin cytoplasmic domain

```
H_sapiens      KLLVTIHDRREFAKFQSERSRARYEMASNPLYRKPISTHTVDFTFNKFNKSYNGTVD 799
M_musculus     KLLVTIHDRREFAKFQSERSRARYEMASNPLYRKPISTHTVDFAFNKFNKSYNGSV- 798
B_taurus       KLLVTIHDRREFAKFQSERSRARYEMASNPLYRKPISTHTVDFTFNKFNKSYNGTVD 800
P_cynocephalus KLLVTIHDRREFAKFQSERSRARYEMASNPLYRKPISTHTVDFTFNKFNKSYNGTVD 655
X_laevis       KLLVTIHDRREFSRFQSDRSRARYEMASNPLYRPAVSTHNVDEMYNMLSKSYNGTT- 802
D_rerio        KLVITVHDRREFARFQSARSRARYEMASNPVYKRSVPMET-DFDMHGIK-SLNGGVH 802
               **::*:******::*** ************:*:  :  ..     : : ** ** .
```

b

β-integrin cytoplasmic domain

```
ITGB5   KLLVTIHDRREFAKFQSERSRARYEMASNPLYRKPISTHTVDFTFNKFNKSYNGTVD--------- 799
ITGB1   KLLMIIHDRREFAKFEKEKMNAKWDTGENPIYKSAVTTVV--------NPKYEGK----------- 798
ITGB2   KALIHLSDLREYRRFEKEKLKSQWNN-DNPLFKSATTTVM--------NPKFAES----------- 769
ITGB3   KLLITIHDRKEFAKFEEERARAKWDTANNPLYKEATSTFT--------NITYRGT----------- 788
ITGB6   KLLVSFHDRKEVAKFEAERSKAKWQTGTNPLYRGSTSTFK--------NVTYKHREKQKVDLSTDC 788
ITGB7   RLSVEIYDRREYSRFEKEQQQLNWKQDSNPLYKSAITTTI--------NPRFQEADSPTL------ 798
        :  : : * :*  :*: *: ..:.   **:::   :*          *
```

c

Position	Residue	Peptide	Predicted Kinases
766	Y	SERSRARYEMASNPL	Src, InsR, EGFR
774	Y	EMASNPLYRKPISTH	Src, InsR, EGFR,Abl2, Itk, Ptk6
794	Y	FNKFNKSYNGTVD	Src, InsR, EGFR, PDGFRa, Fes, Syk, Ptk6

d

		Y766	Y774	Y794
Tool	GPS 5.0		■	■
	Netphos 3.0		■	
	PhosphoSitePlus	■	■	

Supplementary Figure 13. *In silico* analysis of β5-integrin. a, Sequence alignment of cytoplasmic domain of different β5-integrin orthologues. Tyrosine residues are marked in green. Symbols: i) *****, single fully conserved residue; ii) **:** , conservative; iii) **.** , noneconservative. **b,** Sequence alignment of cytoplasmic domain of different β-integrin subfamily members. Symbols as in (a). **c,** Bioinformatic prediction of the possible protein kinases involved in the posttranslational modification of the β5-integrin cytoplasmic domain. The phosphopeptides identified by Netphos 3 and GPS 5 are indicated with the putative modified residues in magenta; the residue position is indicated, as well as the protein kinases most likely involved in the catalysis of the ATP phosho-transfer reaction. **d,** β5-integrin phosphorylation sites prediction. Tyrosine residues present in the β5-integrin cytoplasmic domain are listed with their sequence position indicated. Residues colored with magenta were predicted to be phosphorylated by the indicated bioinformatic tool.

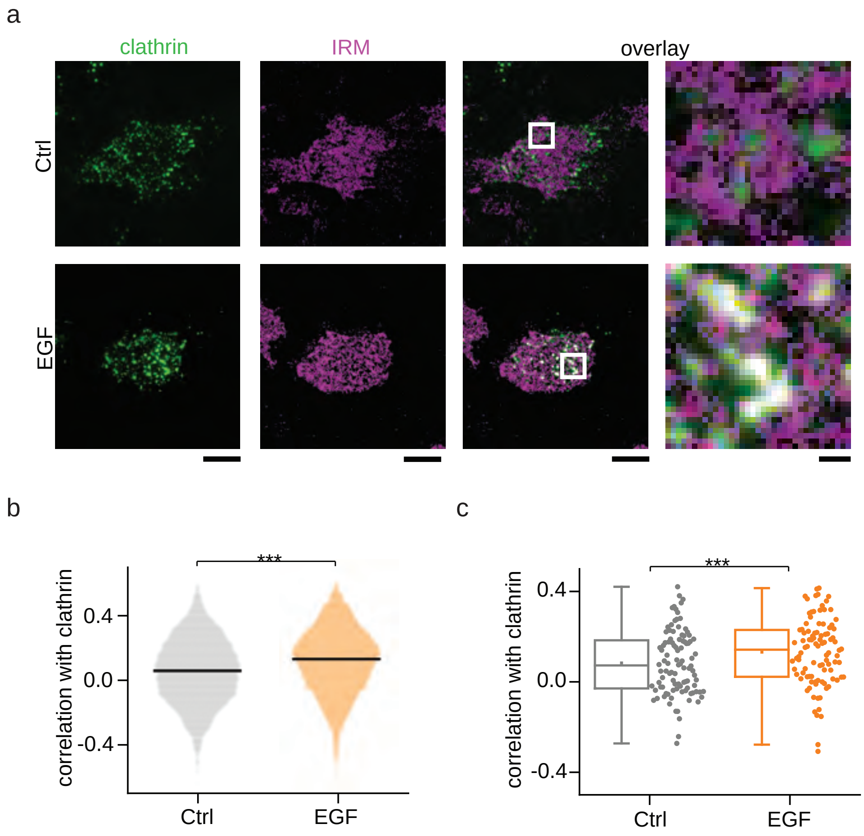

Supplementary Figure 14. EGF increases cell adhesion at clathrin-coated sites. a, Representative TIRF and IRM images of unroofed HSC3 WT transfected with GFP-CLCa before (Ctrl) or after 50 ng/mL EGF stimulation for 15 min. IRM images are inverted and shown in magenta. Scale bars are 10 µm and 1 µm in magnified insets. **b,** Automated correlation analysis of (**a**). Violin plots show median and correlation of all clathrin spots analyzed, ****P*= 8.19×10^{-66}. **c,** Automated correlation of (**a**). Dots show the mean correlation value of every cell analyzed, ****P*=0.02. Box plots show median extended from 25th to 75th percentiles, mean (square) and minimum and maximum data point whiskers with a coefficient value of 1.5. Significance was tested by a two-tailed *t*-test. N=5 biologically independent experiments with consistent results in (**a**-**c**). N_{Ctrl}= 100 cells – 6080 spots; N_{EGF}=98 cells – 6504 spots examined over the indicated independent experiments.

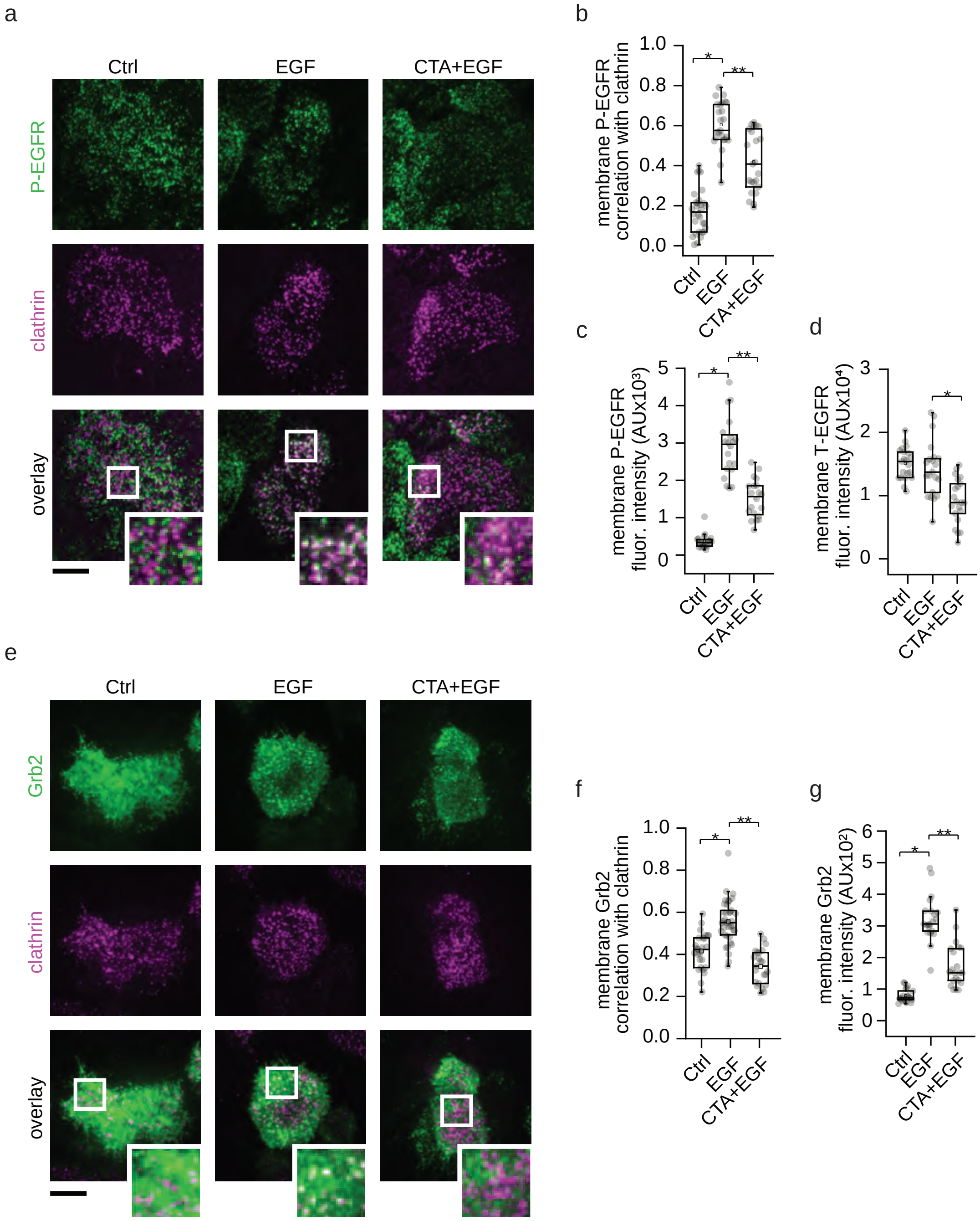

Supplementary Figure 15. Flat clathrin lattices partition sustained signals at the plasma membrane. a, Representative TIRF images of control (Ctrl) unroofed genome-edited HSC3 cells expressing EGFR-GFP transfected with mScarlet-CLCa and immunolabeled with anti-phospho EGFR (P-EGFR) coupled to Alexa 647, treated with 50 ng/mL EGF alone (EGF) or in the presence of 10 µM cilengitide acid (CTA+EGF). **b,** Automated correlation analysis of (**a**). **P*= 9.72×10^{-23}, ***P*=3.13×10^{-6}. N_{Ctrl}=30 cells–2724 spots, N_{EGF}=25 cells–2113 spots, $N_{CTA+EGF}$=23 cells–2223 spots. **c,** Fluorescence intensity measurements of the signal from P-EGFR. **P*=7.23×10^{-17}, ***P*=9.42×10^{-8}. N_{Ctrl}=21 cells, N_{EGF}=20 cells, $N_{CTA+EGF}$=20 cells. **d,** Fluorescence intensity measurements of the signal from total EGFR-GFP (T-EGFR). **P*=3.09×10^{-4}. N_{Ctrl}=21 cells, N_{EGF}=21 cells, $N_{CTA+EGF}$=21 cells. **e,** Representative TIRF images of HSC3 WT cells transfected with mScarlet-CLCa and Grb2-GFP before (Ctrl) or after 15 min EGF stimulation. **f,** Automated correlation analysis of (**e**). **P*=1.18×10^{-7}, ***P*=1.06×10^{-11}. N_{Ctrl}=27 cells–2225 spots, N_{EGF}=38 cells–2588 spots, $N_{CTA+EGF}$=23 cells–2082 spots. **g,** Fluorescence intensity measurements of the signal coming from immunolabeled Grb2. **P*=4.53×10^{-18}, ***P*=3.26×10^{-8}. N_{Ctrl}=21 cells, N_{EGF}=21 cells, $N_{CTA+EGF}$=21 cells. Scale bars are 10 µm; insets are 7.3x7.3 µm square. Dot box plots show median extended from 25th to 75th percentiles, mean (square) and minimum and maximum data point whiskers with a coefficient value of 1.5. N=4 biologically independent experiments with consistent results in (**b,f**) and 3 in (**c,d,f**). Significance was tested by a two-tailed *t*-test. AU, fluorescence arbitrary units.

Supplementary Table 1. Table of information about plasmids generated in our study using In-Fusion HD Cloning Plus (Clonetech, 638911).

Plasmid name	N-terminal protein	C-terminal protein	Mutation	Primer sequence	Plasmid template
EGFR-mScarlet	epidermal growth factor receptor	mScarlet-N	none	AGCGGCCGCGACTCTAGATC	EFGR-GFP Addgene #32751
				GGTGGCGACCGGTGGATC	EFGR-GFP Addgene #32751
				CCACCGGTCGCCACCATGGTGAGCAAGGGCGAGG	mScarlet-N Addgene #85067
				AGAGTCGCGGCCGCTTTACTTGTACAGCTCGTCCATGCC	mScarlet-N Addgene #85067
mScarlet-CLCa	mScarlet-N	clathrin light chain a	none	CGGAGCGAGCTCTTCGGGGAGCCACCATGGTGAGCAAGG	GFP-CLCa
				TGAGTCCGGCCTTGTACAGCTCGTCCATGCC	GFP-CLCa
				GCTGTACAAGGCCGGACTCAGAGCACGAGC	mScarlet-N Addgene #85067
				CTAGAGTCGCGGCCGCTTTATCAGTGCACCAGGGGGGC	mScarlet-N Addgene #85067
β3-integrin-GFP	integrin beta 3	enhanced green fluorescent protein	none	CCGCGGGCCCGGGATCCA	EFGR-GFP Addgene #32751
				CGCTGCTCCCCGAAGAGCT	EFGR-GFP Addgene #32751
				CTTCGGGGAGCAGCGATGCGAGCGCGGCCGCGGCCC	Beta-3-integrin Addgene #27289
				ATCCCGGGCCCGCGGAGTGCCCCGGTACGTGATATTGG	Beta-3-integrin Addgene #27289
β5-integrin-dC-GFP	integrin beta 5	enhanced green fluorescent protein	deletion 743-799 amino acids	CCGCGGGCCCGGGATCCA	β5-integrin-GFP
				CGCTGCTCCCCGAAGAGCT	β5-integrin-GFP
				CTTCGGGGAGCAGCGATGCCGCGGGCCCCGGCG	β5-integrin-GFP
				ATCCCGGGCCCGCGGGACAAGCAGCTTCCAGATAGCC	β5-integrin-GFP
β5-integrin-3YF-GFP*	integrin beta 5	enhanced green fluorescent protein	Tyr766, 774, 794Phe	AAATCCATTATTTAGAAAGCCTATCTCCACGCACACTG	β5-integrin-GFP
				GAAGCCATTTCAAAGCGGGCCCTGGATCGCTC	β5-integrin-GFP
				AACAAATCCTTTAATGGCACTGTG	β5-integrin-GFP
				GAACTTGTTGAAGGTGAAG	β5-integrin-GFP
β5-integrin-3YE-GFP*	integrin beta 5	enhanced green fluorescent protein	Tyr766, 774, 794Glu	AAATCCATTAGAAAGAAAGCCTATCTCCACGCACACTG	β5-integrin-GFP
				GAAGCCATTTCTTCGCGGGCCCTGGATCGCTC	β5-integrin-GFP
				CAACAAATCCGAAAATGGCACTG	β5-integrin-GFP
				AACTTGTTGAAGGTGAAG	β5-integrin-GFP
β5-integrin-2SA-GFP*	integrin beta 5	enhanced green fluorescent protein	Ser759, 762Ala	CGAGCTAGGGCCCGCTATGAAATG	β5-integrin-GFP
				CTCAGCCTGAAACTTTGCAAACTCCC	β5-integrin-GFP

* indicates plasmid generated using Q5 Site-Directed Mutagenesis Kit (New England Biolabs, E0554S).

Supplementary Table 2. Table showing primers used for the identification of the plasmids in our study.

Primer name	Sequence	Company
CMV-F	CGCAAATGGGCGGTAGGCGTG	Psomagen
SV40-pArev	CCTCTACAAATGTGGTATGG	Psomagen
EGFP-NR*	CGTCGCCGTCCAGCTC	Psomagen

*Primer used in plasmids coding for GFP fusions.
