# Supplementary material for: Dual clathrin and integrin signaling systems regulate growth factor receptor activation
Source: Nat Commun. 2022 Feb 16;13:905. doi: 10.1038/s41467-022-28373-x (PMC8850434; doi:10.1038/s41467-022-28373-x)
Supplement: Supplementary file 3 — Reporting Summary [file 41467_2022_28373_MOESM3_ESM.pdf]

nature research

Corresponding author(s): Justin W. Taraska
Last updated by author(s): 2020/12/03

Reporting Summary

Nature Research wishes to improve the reproducibility of the work that we publish. This form provides structure for consistency and transparency in reporting. For further information on Nature Research policies, see our Editorial Policies and the Editorial Policy Checklist.

Statistics

For all statistical analyses, confirm that the following items are present in the figure legend, table legend, main text, or Methods section.

n/a	Confirmed	
☐	☒	The exact sample size (*n*) for each experimental group/condition, given as a discrete number and unit of measurement
☒	☐	A statement on whether measurements were taken from distinct samples or whether the same sample was measured repeatedly
☐	☒	The statistical test(s) used AND whether they are one- or two-sided<br>*Only common tests should be described solely by name; describe more complex techniques in the Methods section.*
☒	☐	A description of all covariates tested
☐	☒	A description of any assumptions or corrections, such as tests of normality and adjustment for multiple comparisons
☐	☒	A full description of the statistical parameters including central tendency (e.g. means) or other basic estimates (e.g. regression coefficient) AND variation (e.g. standard deviation) or associated estimates of uncertainty (e.g. confidence intervals)
☐	☒	For null hypothesis testing, the test statistic (e.g. *F*, *t*, *r*) with confidence intervals, effect sizes, degrees of freedom and *P* value noted<br>*Give P values as exact values whenever suitable.*
☒	☐	For Bayesian analysis, information on the choice of priors and Markov chain Monte Carlo settings
☒	☐	For hierarchical and complex designs, identification of the appropriate level for tests and full reporting of outcomes
☐	☒	Estimates of effect sizes (e.g. Cohen's *d*, Pearson's *r*), indicating how they were calculated

Our web collection on statistics for biologists contains articles on many of the points above.

Software and code

Policy information about availability of computer code

Data collection	Andor IQ2, Nikon (NIS) Elements AR version 5.2, SerialEM 3.8 and IMOD freeware 4.0.26, ImageJ 1.53n, Netphos 3.0, and Group-based prediction system GPS 3.0 software were used to collect the data.
Data analysis	Manual segmentation and morphometry of PREM data and TIRFM fluorescence intensity analysis was done using ImageJ with common tools, such as length and area measurements and intensity profiles. Correlation analysis was done using Matlab code developed and previously reported by our group (Larson et al., 2014) and now available at https://doi.org/10.25444/nhlbi.17159351. Statistical tests were run using OriginPro 2015. Subsequent image processing and figure preparation was done by Adobe Photoshop 2020 and Illustrator 2020.

For manuscripts utilizing custom algorithms or software that are central to the research but not yet described in published literature, software must be made available to editors and reviewers. We strongly encourage code deposition in a community repository (e.g. GitHub). See the Nature Research guidelines for submitting code & software for further information.

Data

Policy information about availability of data

All manuscripts must include a data availability statement. This statement should provide the following information, where applicable:

- Accession codes, unique identifiers, or web links for publicly available datasets
- A list of figures that have associated raw data
- A description of any restrictions on data availability

The protein sequences were consulted in Uniprot database (http://www.uniprot.org/blast/).The raw data generated in this study has been deposited in Figshare at https://doi.org/10.25444/nhlbi.c.5405490. The remaining data are available in the Article or Supplementary Information files. The processed data is available in Supplementary information/Source data provided with this paper.

Field-specific reporting

Please select the one below that is the best fit for your research. If you are not sure, read the appropriate sections before making your selection.

☒ Life sciences ☐ Behavioural & social sciences ☐ Ecological, evolutionary & environmental sciences

For a reference copy of the document with all sections, see nature.com/documents/nr-reporting-summary-flat.pdf

Life sciences study design

All studies must disclose on these points even when the disclosure is negative.

Sample size	No statistical method was used to predetermine sample size because no human or animal subjects were used in this study. Sample size was chosen based on previous experience and standards in the field. For PREM images, the data obtained allowed the analysis of hundreds of individual clathrin structures in multiple cell membranes from biologically independent experiments as we have reported before (see Sochacki et al., 2021; Prasai et al., 2021, Roberts et al., 2020). For TIRF and IRM correlation, more than 1000 spots across multiple cells in at least three independent experiments per condition were analyzed (see Larson et al., 2014, Trexler et al., 2016, Stephens et al., 2020). At least 18 cells from four independent experiments per condition were analyzed for fluorescence intensity measurements. For phosphorylation assays, three biologically independent experiments were performed as reported before (see Tai et al., 2011). High significance values indicate that samples sizes were more than sufficient.
Data exclusions	No reproducible data was excluded from the experiment. However, data resulted from technical errors were excluded for data analysis. For example, if the unroofing procedure resulted in unsatisfactory exposure of the cell interior, thus precluding visualization of structures of interest, such experiments were discarded. These exclusion criteria were pre-established.
Replication	Repetitive biologically independent experiments were done to confirm consistency of results. All attempts at replication were successful for those experiments. Sample sizes (number of cells, structures or spots analyzed) and number of biologically independent experiments with consistent results are indicated in the corresponding figure legends.
Randomization	Not done in this study. In each experiments, there were defined groups e.g. control vs. stimulated, or control vs. specific drug, and randomization was not necessary.
Blinding	Data collection for fluorescent imaging purposes involved selection of random fields of view, the parameters we measured (correlation with thousands of clathrin spots and fluorescence intensity) are not clearly distinguished by the human eye. This implies that the experimenters were inherently blinded to the outcome of each experiment, and no other procedures were necessary. Our analyses relied on automated procedures which are not influenced by the nature of the sample. For PREM manual segmentation, the quantitative analysis was performed by MAAM and KAS with consistent results. We have previously demonstrated that segmentation by independent individuals yields similar results (See Sochacki et al. 2021).

Reporting for specific materials, systems and methods

We require information from authors about some types of materials, experimental systems and methods used in many studies. Here, indicate whether each material, system or method listed is relevant to your study. If you are not sure if a list item applies to your research, read the appropriate section before selecting a response.

Materials & experimental systems

n/a	Involved in the study	
☐	☒	Antibodies
☐	☒	Eukaryotic cell lines
☒	☐	Palaeontology and archaeology
☒	☐	Animals and other organisms
☒	☐	Human research participants
☒	☐	Clinical data
☒	☐	Dual use research of concern

Methods

n/a	Involved in the study	
☒	☐	ChIP-seq
☒	☐	Flow cytometry
☒	☐	MRI-based neuroimaging

Antibodies

Antibodies used	Anti-Clathrin Heavy Chain monoclonal antibody X22 (Thermo-Fisher, MA1-065, Lot VH307789), anti-Phospho-EGF Receptor (Tyr1068) (D7A5) XP® 331 Rabbit mAb (Cell Signaling, 3777, Lot 16), anti-Grb2 Y237 (Abcam, 32037,Lot GR3219704), GAPDH (D16H11) rabbit mAb (HRP conjugated) (Cell Signaling, 8884, Lot 3), EGFR (D38B1) rabbit mAb (Cell Signaling, 4267, Lot 19), β5-integrin (D24A5) rabbit mAb (Cell Signaling, 3629, Lot 1), Src (Thermo Fisher, 14H2L20, Lot UC2735351) rabbit mAb. Secondary antibody conjugated with Alexa Fluor 647 (Invitrogen, anti334 mouse A21237, anti-rabbit A21246).
Validation	Only previously validated antibodies for the relevant assays were used.

Mouse anti-clathrin heavy chain (Invitrogen, #MA1-065, Lot VH307789) has been previously used in our previous publications (Sochacki et al., 2017) and validated by the manufacturer (more information is available at https://www.thermofisher.com/order/genome-database/dataSheetPdf?producttype=antibody&productsubtype=antibody_primary&productId=MA1-065&version=121)
Anti-Phospho-EGF Receptor (Tyr1068) (D7A5) XP® 331 Rabbit mAb (Cell Signaling, #3777, Lot 16) has been previously validated the manufacturer (more infornation is available at https://www.cellsignal.com/datasheet.jsp?productId=3777&images=1) and cited 539 times.
Anti-Grb2 Y237 (Abcam, #32037, Lot GR3219704) has been previously validated by the manufacturer (more information is available at https://www.abcam.com/grb2-antibody-y237-ab32037.html) and cited 19 times.
GAPDH (D16H11) rabbit mAb (HRP conjugated) (Cell Signaling, 8884, Lot 3) was validated by the manufacturer (https://www.cellsignal.com/datasheet.jsp?productId=8884&images=1) and has been cited 55 times.
EGFR (D38B1) rabbit mAb (Cell Signaling, 4267, Lot 19) was previously validated by the manufacturer and does not cross-react with other proteins of the ErbB family (https://www.cellsignal.com/datasheet.jsp?productId=4267&images=1) and has been cited 740 times according to the manufacturer.
β5-integrin (D24A5) rabbit mAb (Cell Signaling, 3629, Lot 1) was validated by the manufacturer to detect endogenous levels of total integrin β5 protein(https://www.cellsignal.com/datasheet.jsp?productId=3629&images=1) and has been cited 19 times.
Src (14H2L20, Lot UC2735351) rabbit mAb was verified by Knockout to ensure that the antibody binds to the antigen stated (Thermo Scientific, 701396) https://www.thermofisher.com/antibody/product/SRC-Antibody-clone-14H2L20-Recombinant-Monoclonal/701396).
All antibodies has been tested and guaranteed by costumers in the literature.

Eukaryotic cell lines

Policy information about cell lines

Cell line source(s)	Wild-type HSC-3 (human oral squamous carcinoma) cells were obtained from the JCRB Cell Bank (JCRB0623). Genome-edited HSC-3 cells expressing endogenous EGFR-GFP were previously reported (Pinilla-Macua et al., 2017) and kindly donated by Dr. Alexander Sorkin (University of Pittsburgh).
Authentication	No
Mycoplasma contamination	All cell lines were routinely tested for the mycoplasma contamination and were negative.
Commonly misidentified lines (See ICLAC register)	No commonly misidentified lines were used in this study.
